# A machine learning-based risk prediction model for early preterm birth: development and prospective validation

**DOI:** 10.3389/fmed.2026.1888022

**Published:** 2026-07-22

**Authors:** Liang He, Kailin Luo, Rong Wang, Gaoying Wang, Jianyi Gao, Jing Wang, Ruirui Dong

**Affiliations:** 1 Affiliated Women's Hospital of Jiangnan University, Jiangnan University, Wuxi, China; 2 Jiangsu Tianyi High School, Wuxi, China

**Keywords:** machine learning, mid-pregnancy, preterm birth, prospective validation, risk prediction

## Abstract

**Introduction:**

Preterm birth (PTB) remains a leading cause of neonatal morbidity and mortality worldwide, yet accurate and clinically applicable risk prediction tools during mid-pregnancy are still limited.

**Methods:**

We developed a machine learning–based model to predict PTB risk using routinely collected clinical and laboratory data obtained during mid-pregnancy. In a derivation cohort, candidate predictors were selected using least absolute shrinkage and selection operator (LASSO) regularization, followed by training and comparison of multiple machine learning algorithms. A light gradient boosting machine (LightGBM) model was selected as the final model. Model performance was evaluated in a temporally independent prospective validation cohort from the same tertiary maternity center in terms of discrimination, calibration, and clinical utility. A screening-oriented risk stratification strategy was applied based on the distribution of predicted probabilities. Model interpretability was assessed using SHapley Additive exPlanations (SHAP) to quantify feature contributions.

**Results:**

In the internal test set of the derivation cohort, LightGBM demonstrated the best discriminative performance, achieving an area under the receiver operating characteristic curve (AUC) of 0.842. In the prospective validation cohort, the model maintained stable discrimination and good calibration. Using a screening-oriented risk stratification approach, women classified as high risk exhibited a substantially higher incidence of PTB compared with the intermediate- and low-risk groups (31.8% vs. 6.0% and 2.5%, respectively), with clear separation of cumulative PTB incidence across gestational age. Decision curve analysis demonstrated a consistent net benefit across a range of low threshold probabilities. SHAP analysis revealed that inflammatory markers, liver-related biochemical indices, pregnancy complications, and nutritional indicators were key contributors to model predictions, supporting clinical interpretability.

**Conclusions:**

We developed a machine learning–based model for mid-pregnancy PTB risk prediction and evaluated it in a temporally independent prospective validation cohort from the same tertiary maternity center. The model demonstrated stable discrimination, good calibration, meaningful risk stratification, and transparent interpretability, supporting its potential use as a screening-oriented decision-support tool for targeted antenatal surveillance.

## 1 Introduction

Preterm birth (PTB), defined as delivery before 37 completed weeks of gestation, remains a leading cause of neonatal morbidity and mortality worldwide and imposes a substantial burden on families and healthcare systems (1). Infants born preterm are at increased risk of short- and long-term complications, including respiratory distress, neurodevelopmental impairment, and chronic health conditions (2–5). Despite advances in perinatal care, the global incidence of PTB has not declined substantially (6), underscoring the need for effective strategies to identify women at increased risk during pregnancy.

Early identification of women at increased risk of PTB is essential for optimizing antenatal surveillance and preventive strategies; however, accurate prediction remains challenging due to the heterogeneous etiology of PTB and the complex interplay of maternal, fetal, and environmental factors. Recent studies have explored machine learning and deep learning approaches for PTB prediction using diverse data sources, including structured electronic health records, antenatal-care cohort data, routinely available biomarkers, and multi-omics profiles. Models developed from electronic health records in China (7), antenatal-care data from population-based cohorts (8), and repeated prenatal-visit information from electronic medical records (9) further support the feasibility of applying clinically available predictors to scalable PTB risk assessment across pregnancy. Large retrospective cohort analyses based on routinely collected clinical data have also demonstrated that advanced algorithms, including transformer-based models, can achieve moderate discriminative performance and high negative predictive value, supporting their potential role in risk screening (10). Other investigations have focused on biomarker-driven models, such as those derived from maternal serum proteomics, which reported high predictive accuracy in selected cohorts but relied on specialized assays that may limit scalability and routine clinical implementation (11). More recently, transformer-based architectures integrating multi-omics data, including cell-free DNA and RNA, have shown promising improvements in predictive performance, highlighting the potential of artificial intelligence–driven data fusion for precision obstetrics (12). Nevertheless, many existing models are constrained by retrospective designs, limited prospective validation, and insufficient evaluation of calibration and clinical utility, which hampers their translation into real-world mid-pregnancy risk screening.

Importantly, from a clinical perspective, prediction models for PTB are primarily intended for risk screening rather than definitive diagnosis or causal inference. In screening-oriented settings, model performance should emphasize reliable discrimination and calibration to support risk stratification, enabling efficient identification of women at relatively higher risk who may benefit from enhanced surveillance (13). In addition, transparent model interpretability is essential to support clinical trust and understanding. Model-agnostic explanation methods, such as SHapley Additive exPlanations (SHAP), provide insight into the relative contribution of individual features to model predictions while accommodating non-linear effects and interactions inherent to machine learning models (14). Accordingly, prospective validation and formal evaluation of clinical usefulness, including decision curve analysis, are critical for determining whether prediction models can meaningfully support real-world antenatal care.

**Figure 1:**
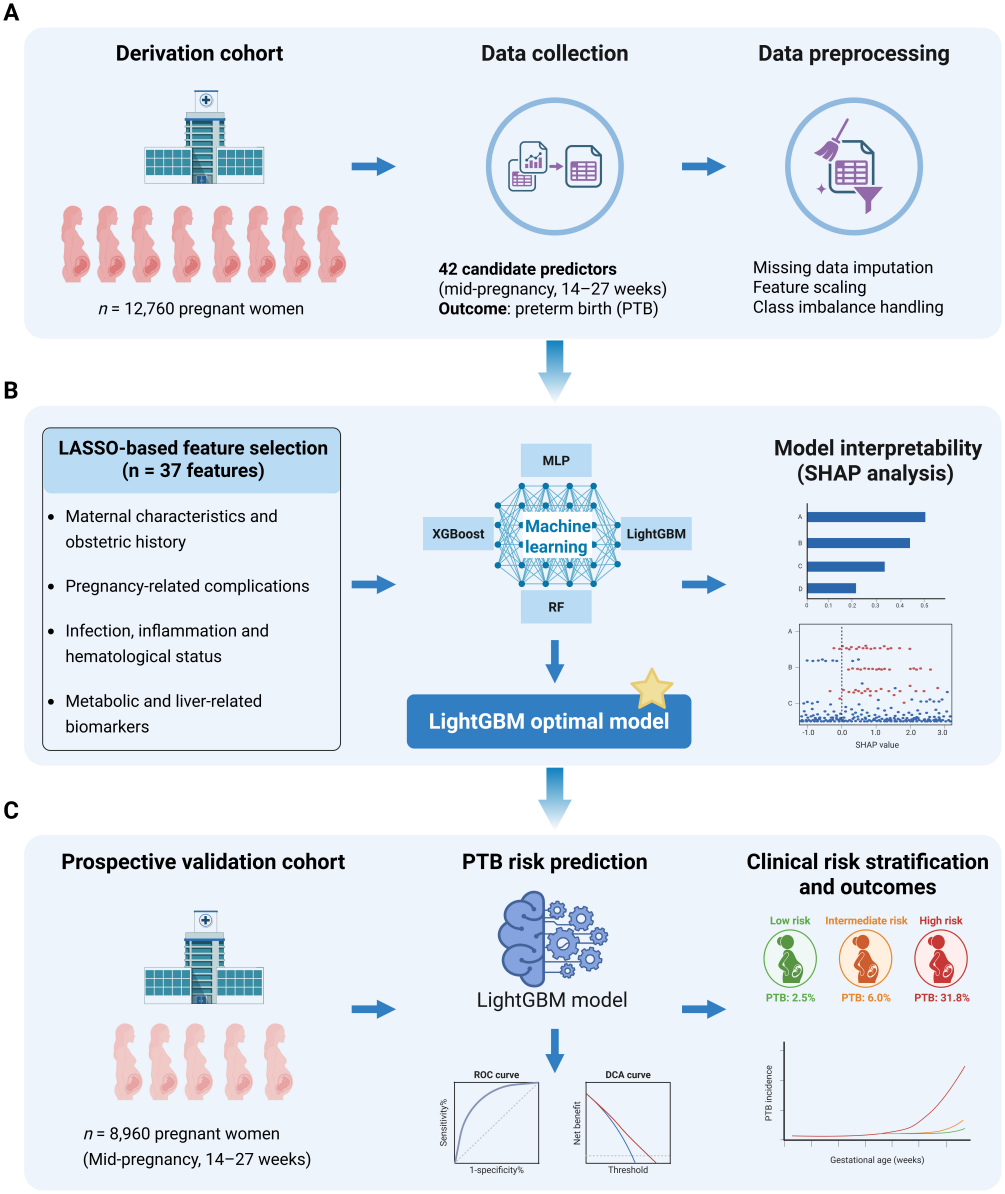
Study workflow for model development and validation. **(A)** Derivation cohort, data collection, and preprocessing during mid-pregnancy (14–27 weeks). **(B)** Feature selection, model development, and interpretability analysis. **(C)** Prospective validation and clinical risk stratification for preterm birth. LASSO, least absolute shrinkage and selection operator. LightGBM, light gradient boosting machine; MLP, multilayer perceptron; RF, random forest; XGBoost, extreme gradient boosting; SHAP, SHapley Additive exPlanations; PTB, preterm birth.

In this study, we developed and prospectively validated a machine learning–based model for mid-pregnancy risk prediction of PTB using routinely collected clinical and laboratory data. As illustrated in Figure 1, candidate predictors collected during mid-pregnancy underwent standardized preprocessing and feature selection using least absolute shrinkage and selection operator (LASSO) regularization, followed by a systematic comparison of multiple machine learning algorithms. A light gradient boosting machine (LightGBM) model was selected as the optimal model and further evaluated in a temporally independent prospective validation cohort from a subsequent time period at the same tertiary maternity center with respect to discrimination, calibration, clinical utility, and risk stratification performance. To enhance transparency and clinical interpretability, SHAP analysis was used to quantify the relative contribution of individual features to model predictions. By integrating model interpretability and prospective validation within a screening-oriented framework, this study aimed to establish a clinically applicable approach for PTB risk stratification during mid-pregnancy to support more targeted antenatal surveillance.

## 2 Methods

### 2.1 Study population

**Figure 2:**
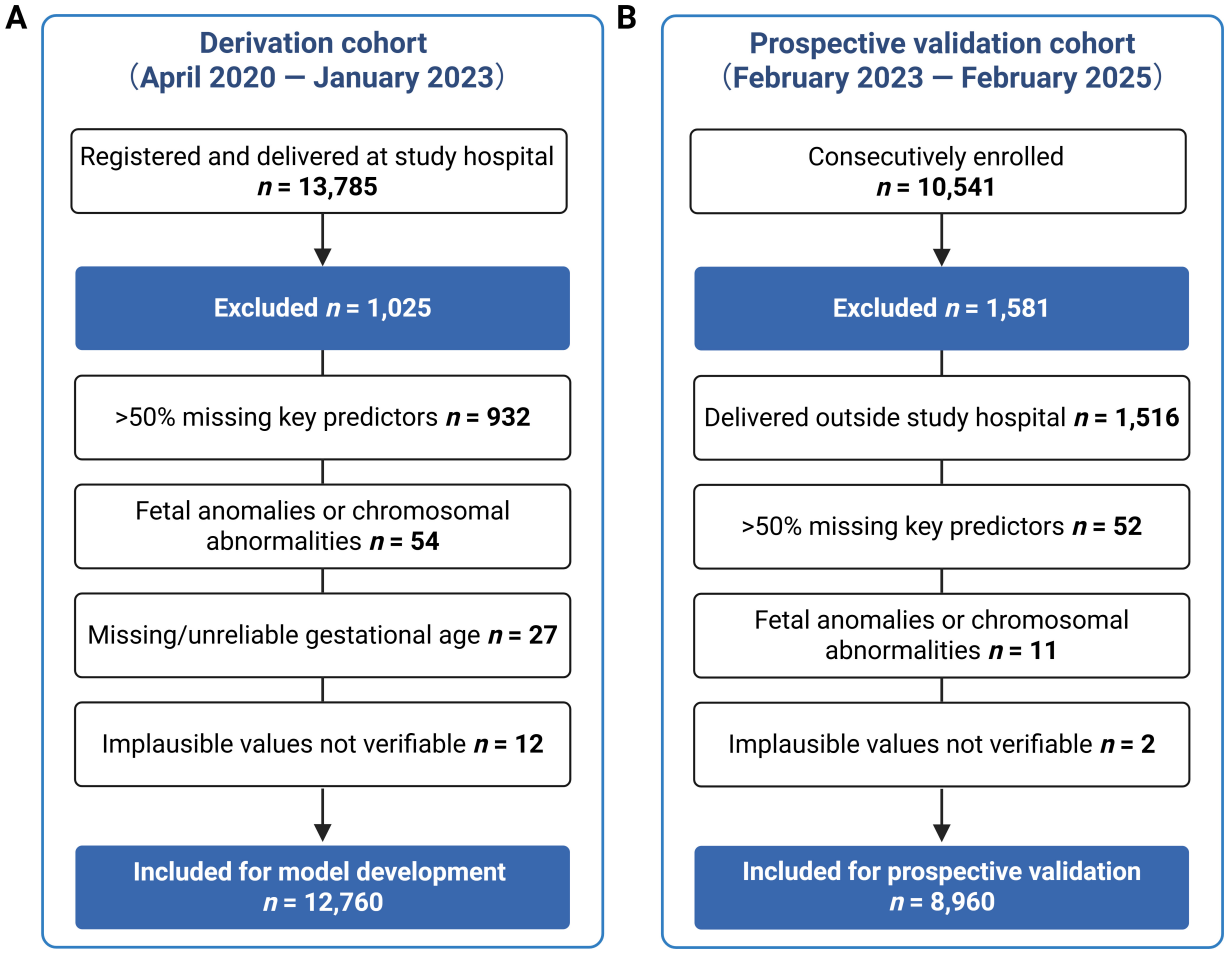
Flow diagram of participant inclusion and exclusion. **(A)** Derivation cohort (April 2020–January 2023). **(B)** Prospective validation cohort (February 2023–February 2025). Eligible pregnant women were screened according to predefined exclusion criteria, and the final cohorts were included for model development and prospective validation, respectively.

This study was conducted at the Affiliated Women's Hospital of Jiangnan University (Wuxi Maternity and Child Health Care Hospital), a tertiary maternity hospital in China, and included two independent cohorts: a derivation cohort for model development and internal evaluation, and a prospective temporal validation cohort from a subsequent time period at the same tertiary maternity center. The derivation cohort comprised pregnant women who registered for antenatal care and delivered at the study hospital between April 2020 and January 2023. A total of 13,785 pregnancies were initially identified. Women were excluded if they had more than 50% missing key predictors, fetal anomalies or chromosomal abnormalities, missing or unreliable gestational age, or implausible clinical values that could not be verified. After applying these exclusion criteria, 12,760 women were included for model development (Figure 2A).

The prospective validation cohort consisted of consecutively enrolled pregnant women who received antenatal care during mid-pregnancy and delivered between February 2023 and February 2025. Among 10,541 eligible pregnancies, women were excluded if delivery occurred outside the study hospital, if more than 50% of key predictors were missing, if fetal anomalies or chromosomal abnormalities were present, or if implausible values could not be verified. The final prospective validation cohort included 8,960 women (Figure 2B). This study was approved by the Institutional Review Board of the Affiliated Women's Hospital of Jiangnan University [Wuxi Maternity and Child Health Care Hospital Ethical Review (2020) No. 011]. All participants provided written informed consent before enrollment. The intended target population was pregnant women receiving routine mid-pregnancy antenatal care at the study tertiary maternity hospital and planning delivery within the same care system. Both singleton and multiple pregnancies were eligible, and the study cohort was not restricted to a preselected high-risk obstetric population.

### 2.2 Data collection

#### 2.2.1 Data sources and candidate predictors

Clinical and laboratory data were collected from the electronic medical record system of the Affiliated Women's Hospital of Jiangnan University. Candidate predictors were obtained during mid-pregnancy (14–27 weeks of gestation), corresponding to routine antenatal visits. A total of 42 candidate predictors were initially considered, including maternal demographic characteristics, obstetric history, pregnancy-related complications, and routinely measured laboratory indices. Clinical variables comprised maternal age, anthropometric measures, gravidity and parity, mode of conception, and pre-existing or pregnancy-related conditions documented during antenatal care. Laboratory variables included hematological indices, biochemical markers, inflammatory and infection-related parameters, and metabolic and liver-related biomarkers, all measured as part of standard clinical practice.

Gestational age was determined based on the best obstetric estimate, integrating last menstrual period and early ultrasound findings according to routine clinical protocols. Outcome data, including gestational age at delivery and PTB status, were extracted from delivery records after childbirth. PTB was defined as delivery before 37 completed gestational weeks. Overall PTB was prespecified as the primary outcome because the model was intended as a screening-oriented risk stratification tool for routine mid-pregnancy antenatal care. In this clinical context, the initial objective is to identify women at increased risk of delivery before 37 completed gestational weeks, regardless of the eventual pathway leading to preterm delivery. The original dataset did not contain a reliable and consistently structured variable distinguishing spontaneous PTB from medically indicated PTB. All data were collected retrospectively for the derivation cohort and prospectively for the validation cohort using identical variable definitions and standardized data extraction procedures to ensure consistency across cohorts.

#### 2.2.2 Prediction landmark and temporal availability of predictors

To avoid temporal leakage and to reflect a clinically realistic prediction scenario, the prediction landmark was prespecified before model development. For each participant, the prediction landmark was defined as the date of the routine antenatal visit within the 14–27 completed gestational week window at which the predictor set was assembled. This date represented the clinical time point at which the model would be applied, using only information already available to clinicians. Only information available on or before this landmark was eligible for use as a predictor. The timing and ascertainment rules for all predictor domains are summarized in Supplementary Table S1.

Laboratory indicators were extracted from routine tests performed within the 14–27 completed gestational week prediction window. To mirror information availability in real-world antenatal care, the eligible value for each laboratory variable was defined as the most recent result available on or before the individual prediction landmark. If multiple measurements were recorded before the landmark, the measurement closest to the landmark was selected. Measurements obtained after the landmark were excluded from predictor definition. If no eligible measurement was available before or at the landmark, the variable was considered missing and processed according to the prespecified imputation strategy described below.

For pregnancy-related complications that could arise later in gestation, including gestational diabetes mellitus, intrahepatic cholestasis of pregnancy, preeclampsia, and pregnancy-induced hypertension, diagnosis dates were reviewed in relation to the prediction landmark. These variables were coded as present only if the diagnosis had been documented on or before the landmark. Diagnoses first recorded after the landmark were not used to define predictor status and were coded as absent at the time of prediction. Information recorded after the prediction landmark was used only for outcome ascertainment. The temporal verification results for these pregnancy-related complications are provided in Supplementary Table S2.

### 2.3 Data processing

All candidate predictors were processed using a prespecified pipeline to ensure consistency across models. Unless otherwise stated, data processing steps were implemented within the derivation cohort to avoid information leakage, and the same transformations were subsequently applied to the prospective validation cohort.

#### 2.3.1 Missing value imputation

Before imputation, the extent of missingness was summarized for each candidate predictor and reported in Supplementary Table S3. Categorical predictors had no missing values. For continuous predictors, the proportion of missing values varied across variables. The highest missingness was observed for lipid and lipoprotein-related markers, including Lp(a), TC, TG, HDL, LDL, ApoA1, and ApoB, each with approximately 49% missing values. CRP had 7.0% missingness, whereas most hematological, liver function, protein/nutritional, and maternal anthropometric variables had low missingness, generally below 1.5%.

For model development, missing values in continuous variables were imputed using median values derived exclusively from the training set of the derivation cohort. The same imputation parameters were then applied unchanged to the internal test set and the prospective validation cohort to avoid information leakage. Missingness indicators were not included in the primary model. This decision was made because the intended use of the model was to generate risk predictions from routinely available clinical and laboratory values, whereas missingness patterns may partly reflect institution-specific test-ordering practices, laboratory availability, or care pathways rather than patient biology alone. Nevertheless, we acknowledge that missingness itself may contain predictive information, particularly for variables with higher missingness. Therefore, the distribution of missingness was explicitly reported and considered when interpreting model transportability.

#### 2.3.2 Data standardization

To place continuous predictors on a comparable scale, continuous variables were standardized using z-score normalization (subtracting the mean and dividing by the standard deviation). The mean and standard deviation were calculated exclusively from the training set of the derivation cohort and subsequently applied to transform the internal test set of the derivation cohort as well as the prospective validation cohort. Tree-based models were trained using the same processed dataset.

#### 2.3.3 Handling class imbalance

Given the imbalanced distribution of PTB, cost-sensitive learning strategies were applied during model training. Specifically, class- or sample-weighting approaches were adopted depending on the modeling framework to upweight the minority class (PTB). For logistic regression and LASSO-regularized logistic regression, class weights were applied. For tree-based boosting models [LightGBM and extreme gradient boosting (XGBoost)], class imbalance was handled using the scale_pos_weight parameter. For the multilayer perceptron (MLP) and random forest (RF) models, sample-level weighting was incorporated during training, with higher weights assigned to PTB cases proportional to the inverse of class frequencies.

### 2.4 Model development and feature selection

To develop prediction models for preterm birth, the derivation cohort was randomly divided into a training set (70%) and an internal test set (30%) using stratified sampling to preserve the distribution of PTB in both subsets. As a baseline model and to assess the effect of candidate predictor use before machine learning model development, a multivariable logistic regression model was first constructed using all 42 candidate predictors obtained during mid-pregnancy. Subsequently, LASSO-regularized logistic regression was applied to derive a more parsimonious predictor set. Because the number of candidate predictors was modest and most predictors were clinically relevant, LASSO was used as a regularization-based predictor screening approach to reduce potential overfitting, improve predictor stability in the presence of correlated clinical and laboratory variables, and provide a reproducible input feature set for subsequent machine learning model development. The optimal regularization parameter was determined based on the minimum cross-validated error (λ min).

Using this criterion, 37 predictors were retained for subsequent model development. These included maternal characteristics (maternal age, maternal weight, gravidity = 2, parity = 1, parity ≥ 2, multiple pregnancy, *in vitro* fertilization, obesity, uterine fibroid, and cardiac arrhythmia), pregnancy-related complications (gestational diabetes mellitus, intrahepatic cholestasis of pregnancy, preeclampsia, pregnancy-induced hypertension, and thyroid disease), infection-related variables (group B Streptococcus infection, vaginitis, and hepatitis B virus infection), hematological indices (hemoglobin, neutrophil count, and lymphocyte count), and biochemical and metabolic markers [retinol-binding protein, prealbumin, alpha-L-fucosidase, leucine aminopeptidase, cholinesterase, total bilirubin, direct bilirubin, total protein, albumin, alanine aminotransferase, total cholesterol, triglycerides, low-density lipoprotein cholesterol, apolipoprotein A1, apolipoprotein B, and lipoprotein(a)]. The selected features were subsequently used for training and comparison of machine learning models in the derivation cohort. To evaluate whether the LASSO-based feature selection step affected final machine learning performance, we additionally performed a sensitivity analysis by retraining the final LightGBM model using all 42 candidate predictors without LASSO-based preselection.

### 2.5 Machine learning model training and tuning

Based on the selected predictors, four machine learning algorithms, namely random forest (RF), multilayer perceptron (MLP), light gradient boosting machine (LightGBM), and extreme gradient boosting (XGBoost), were trained using the training set of the derivation cohort. Model development and hyperparameter tuning were conducted exclusively within the derivation cohort to avoid information leakage. Hyperparameters for all machine learning models were optimized using random search with 5-fold cross-validation within the training set, and the optimal configurations were selected based on the mean area under the receiver operating characteristic curve across cross-validation folds.

The RF model was constructed as an ensemble of decision trees using bootstrap aggregation, with sample-level weighting applied during training such that PTB cases were assigned higher weights proportional to the inverse of class frequencies. The MLP model was implemented as a feedforward neural network, and cost-sensitive learning was incorporated by applying sample weights during training to mitigate the impact of class imbalance. For LightGBM and XGBoost, gradient boosting decision tree–based classifiers were implemented to capture complex non-linear relationships and interactions among predictors. Class imbalance was addressed by specifying the scale_pos_weight parameter based on the distribution of PTB in the training data. The number of random-search iterations was 30 for RF, 50 for MLP, 30 for LightGBM, and 80 for XGBoost.

Final model performance was evaluated on the internal test set of the derivation cohort. The optimized models were then fixed and subsequently evaluated in the prospective validation cohort without further retraining or parameter adjustment. For the all-predictor sensitivity analysis, the LightGBM model was retrained using all 42 candidate predictors while keeping the same training/testing split, preprocessing procedures, hyperparameters, class-imbalance strategy, and number of boosting iterations as the primary LightGBM model.

All analyses were performed in R version 4.5.1. The main packages used included caret 7.0.1, glmnet 4.1.10, ranger 0.18.0, lightgbm 4.6.0, xgboost 3.2.1.1, nnet 7.3.20, pROC 1.19.0.1, dplyr 1.2.0, ggplot2 4.0.2, Matrix 1.7.4, and data.table 1.18.2.1. Fixed random seeds were used to ensure reproducibility of model development, cross-validation, random-search tuning, validation analyses, and figure generation. Full hyperparameter search spaces, final selected parameters, software versions, random seeds, and SHAP calculation details are provided in Supplementary Tables S4 and S5.

### 2.6 Model performance evaluation

Model performance was evaluated in the held-out internal test set of the derivation cohort and in the temporally independent prospective validation cohort with respect to discrimination, threshold-dependent classification performance, calibration, and clinical utility. Discriminative performance was assessed using receiver operating characteristic (ROC) curves and quantified by the area under the ROC curve (AUC) with 95% confidence intervals (CIs). In addition, given the relatively low incidence of PTB, precision-recall performance was assessed using the area under the precision-recall curve (PR-AUC). Threshold-dependent performance was evaluated using sensitivity, specificity, positive predictive value (PPV), negative predictive value (NPV), and F1-score. In the internal test set, these metrics were calculated using model-specific thresholds determined from the ROC curve. For the prospective validation cohort, threshold-dependent metrics for the final LightGBM model were calculated using the prespecified LightGBM threshold derived from the internal test set, without re-optimizing the threshold in the validation cohort.

Calibration was evaluated using calibration curves, calibration intercept, calibration slope, and the Brier score. Calibration curves were generated to compare predicted and observed PTB risks, and bootstrap bias-corrected calibration curves were used to assess optimism-corrected agreement. The calibration intercept and calibration slope were estimated by fitting a logistic calibration model with the observed PTB status as the outcome and the logit-transformed predicted probability as the predictor. The Brier score was calculated as the mean squared difference between predicted probabilities and observed outcomes, with lower values indicating better overall accuracy of probabilistic prediction.

### 2.7 Clinical utility assessment and risk stratification

Clinical utility was assessed using decision curve analysis (DCA) to quantify the net benefit of the prediction model across a range of threshold probabilities. DCA was performed using continuous predicted probabilities without applying predefined risk group thresholds. The model was compared with default strategies of classifying all women as high risk or none as high risk. For clinical interpretation, we focused primarily on threshold probabilities between 5% and 10%, which were considered relevant to screening-oriented mid-pregnancy PTB risk assessment. This range reflects a setting in which model-triggered actions would mainly involve lower-burden interventions, such as closer surveillance, referral, or individualized counseling, rather than immediate invasive treatment. Threshold probabilities of 5% and 10% correspond to accepting approximately 19 and 9 unnecessary intensified-surveillance interventions, respectively, to identify one additional PTB event. A threshold probability of 15% was additionally summarized as a higher-threshold reference point. To facilitate clinical interpretation, we reported net benefit values and the net reduction in unnecessary intensified-surveillance interventions per 100 women compared with classifying all women as high risk.

Risk stratification was intended as a screening-oriented operational categorization rather than as a diagnostic cutoff. Because no universally accepted probability threshold exists for mid-pregnancy PTB risk stratification, the primary risk stratification thresholds were derived from the predicted probability distribution in the derivation cohort and fixed before application to the prospective validation cohort. Women in the top 15% of predicted probabilities in the derivation cohort were classified as high risk, whereas those in the bottom 50% were classified as low risk; the remaining women were classified as intermediate risk. The corresponding probability cutoffs were then applied unchanged to the prospective validation cohort.

The top 15% threshold was selected to identify a clinically manageable high-risk subgroup enriched for PTB risk who may benefit from intensified surveillance, while avoiding an excessively large high-risk category that would be difficult to implement in routine antenatal care. The bottom 50% threshold was selected to define a large low-risk group in whom unnecessary escalation of care may be avoided. These thresholds were not optimized using outcome information from the prospective validation cohort. To assess the clinical relevance of the proposed risk stratification, observed PTB incidence was compared across risk groups, and cumulative PTB incidence over gestational age was evaluated using time-to-event analysis.

To assess whether the observed risk stratification pattern was sensitive to the selected cutoffs, we performed sensitivity analyses using alternative percentile-based thresholds derived from the derivation cohort and applied unchanged to the prospective validation cohort. Specifically, we varied the high-risk threshold to the top 10% and top 20% while keeping the low-risk threshold at the bottom 50%, and varied the low-risk threshold to the bottom 40% and bottom 60% while keeping the high-risk threshold at the top 15%. For each strategy, observed PTB incidence was calculated across low-, intermediate-, and high-risk groups in the prospective validation cohort. Risk gradients were evaluated using trend tests, and risk ratios comparing the high-risk group with the low-risk group were calculated.

### 2.8 Model explanation

Model interpretability was assessed to facilitate understanding of the contribution of individual predictors to the model output. Feature importance and model explanations were derived using SHAP, a game theory–based approach that provides consistent and locally accurate attribution of feature effects (14). SHAP analysis was performed for the final LightGBM model using the training set of the derivation cohort. SHAP values were calculated to quantify the global contribution of each predictor to model predictions of preterm birth. Global feature importance was summarized by the mean absolute SHAP value across all samples. SHAP dependence plots were further generated to visualize the relationship between predictor values and their corresponding SHAP values, allowing assessment of potential non-linear effects and interactions learned by the model. Model explanation was conducted for interpretative purposes only and did not imply causal relationships or independent associations between predictors and the outcome.

### 2.9 Statistical analysis

All analyses were performed using R software (version 4.5.1). Baseline characteristics were summarized using descriptive statistics. Continuous variables were presented as mean ± standard deviation (SD) or median with interquartile range (IQR), as appropriate, and categorical variables were summarized as number (percentage). Standardized mean differences (SMDs) were reported to quantify the magnitude of differences between groups. No formal statistical hypothesis testing was performed for baseline comparisons, as these analyses were descriptive in nature. To quantify statistical uncertainty in model comparison, AUCs were reported with 95% CIs. Because all candidate models were evaluated in the same internal test set, pairwise comparisons of AUCs between LightGBM and alternative models were performed using paired DeLong tests.

For risk stratification analyses, the incidence of PTB across model-defined risk groups was summarized descriptively, and group differences were assessed using the chi-square test or Fisher's exact test, as appropriate, for descriptive comparison. Time-to-event analysis was further conducted to evaluate differences in cumulative incidence of PTB across risk strata. Gestational age (weeks) was used as the time scale, and PTB was treated as the event of interest. Cumulative incidence curves were estimated using the Kaplan–Meier method, and differences between risk groups were assessed using the log-rank test. *P* values were reported for these comparisons for descriptive purposes only.

## 3 Results

### 3.1 Baseline characteristics of the study population

A total of 12,760 pregnant women were included in the derivation cohort and 8,960 in the prospective validation cohort. Baseline characteristics of the two cohorts are summarized in Table 1. Overall, the distributions of maternal demographic characteristics, obstetric history, pregnancy-related complications, and laboratory indices were broadly comparable between the derivation and validation cohorts.

**Table 1:** Baseline characteristics of pregnant women in the derivation and prospective validation cohorts.

Characteristic	Derivation cohort *n* = 12,760	Prospective validation cohort *n* = 8,960
Maternal age, years	29.5 (26.9, 32.3)	30.0 (27.5, 33.1)
Height, cm	162.0 (158.0, 165.0)	162.0 (158.0, 165.0)
Weight, kg	70.0 (64.5, 77.0)	71.0 (64.0, 78.0)
Body mass index, kg/m^2^	26.9 (24.8, 29.3)	27.0 (24.8, 29.4)
Gravidity, *n* (%)		
1	5,592 (44%)	4,094 (46%)
2	3,562 (28%)	2,420 (27%)
≥3	3,606 (28%)	2,446 (27%)
Parity, *n* (%)		
0	8,113 (64%)	5,877 (66%)
1	4,111 (32%)	2,639 (29%)
≥2	536 (4.2%)	444 (5.0%)
Multiple pregnancy, *n* (%)	319 (2.5%)	324 (3.6%)
Gestational diabetes mellitus, *n* (%)	2,594 (20%)	1,973 (22%)
Intrahepatic cholestasis of pregnancy, *n* (%)	197 (1.5%)	172 (1.9%)
Pregnancy-induced hypertension, *n* (%)	443 (3.5%)	325 (3.6%)
Preeclampsia, *n* (%)	458 (3.6%)	347 (3.9%)
Thyroid disease, *n* (%)	957 (7.5%)	769 (8.6%)
Scarred uterus, *n* (%)	2,166 (17%)	1,457 (16%)
Maternal obesity, *n* (%)	596 (4.7%)	592 (6.6%)
Group B Streptococcus infection, *n* (%)	807 (6.3%)	808 (9.0%)
Vaginitis, *n* (%)	211 (1.7%)	195 (2.2%)
Uterine fibroids, *n* (%)	652 (5.1%)	651 (7.3%)
Cardiac arrhythmia, *n* (%)	156 (1.2%)	39 (0.4%)
*In vitro* fertilization, *n* (%)	884 (6.9%)	587 (6.6%)
Hepatitis B virus infection, *n* (%)	402 (3.2%)	193 (2.2%)
Preterm birth, *n* (%)	896 (7.0%)	800 (8.9%)
Neutrophil count, × 10^9^/L	6.6 (5.3, 8.2)	6.5 (5.3, 8.5)
Lymphocyte count, × 10^9^/L	1.5 (1.2, 1.8)	1.4 (1.2, 1.8)
Hemoglobin, g/L	120.0 (111.0, 128.0)	120.0 (112.0, 128.0)
C-reactive protein, mg/L	2.5 (2.5, 3.9)	2.5 (2.5, 4.9)
Retinol-binding protein, mg/L	34.4 (29.5, 39.7)	35.0 (30.1, 40.2)
Prealbumin, mg/L	202.1 (178.5, 226.7)	200.9 (177.3, 223.7)
α-L-fucosidase, U/L	51.1 (43.6, 59.3)	50.0 (42.3, 57.8)
Leucine aminopeptidase, U/L	238.9 (188.3, 300.6)	244.5 (192.4, 305.9)
Cholinesterase, U/L	5,492.5 (4,841.0, 6,172.0)	5,435.0 (4,777.5, 6,151.4)
Total bilirubin, μmol/L	8.0 (6.6, 9.8)	8.1 (6.6, 9.9)
Direct bilirubin, μmol/L	2.1 (1.7, 2.6)	2.1 (1.7, 2.7)
Total protein, g/L	63.2 (60.1, 66.3)	63.3 (60.2, 66.1)
Albumin, g/L	35.7 (33.8, 37.4)	35.1 (33.3, 36.8)
Alanine aminotransferase, U/L	9.9 (7.7, 13.2)	10.2 (7.8, 13.9)
Aspartate aminotransferase, U/L	17.0 (14.8, 20.2)	16.9 (14.4, 20.1)
Total cholesterol, mmol/L	6.4 (5.7, 7.3)	6.3 (5.6, 7.2)
Triglycerides, mmol/L	3.4 (2.6, 4.4)	3.1 (2.4, 4.2)
High-density lipoprotein cholesterol, mmol/L	2.2 (1.9, 2.5)	2.3 (2.0, 2.5)
Low-density lipoprotein cholesterol, mmol/L	4.1 (3.5, 4.8)	4.0 (3.4, 4.8)
Apolipoprotein A1, g/L	2.2 (2.0, 2.4)	2.2 (2.0, 2.4)
Apolipoprotein B, g/L	1.3 (1.1, 1.5)	1.3 (1.1, 1.5)
Lipoprotein(a), mg/L	79.2 (35.4, 173.8)	94.6 (42.1, 204.6)

Data are presented as median (interquartile range, IQR) for continuous variables and as number (percentage) for categorical variables.

In the derivation cohort, the median maternal age was 29.5 years (IQR, 26.9–32.3), and the median gestational body mass index was 26.9 kg/m^2^ (IQR, 24.8–29.3). Nulliparity accounted for approximately two-thirds of pregnancies, and multiple pregnancy was observed in 2.5% of women. Common pregnancy-related complications included gestational diabetes mellitus (20.0%), thyroid disease (7.5%), and preeclampsia (3.6%). The derivation cohort included 896 PTB events among 12,760 pregnancies, corresponding to an overall PTB incidence of 7.0%. The prospective validation cohort included 800 PTB events among 8,960 pregnancies, corresponding to an incidence of 8.9%. Baseline laboratory parameters, including hematological indices, inflammatory markers, liver function tests, lipid profiles, and nutritional markers, showed similar distributions across cohorts. Minor differences were observed in the prevalence of selected clinical conditions, such as multiple pregnancy and group B Streptococcus infection, reflecting temporal variation between cohorts rather than systematic imbalance.

Within the derivation cohort, baseline characteristics stratified by PTB status are presented in Supplementary Table S6. Compared with women who delivered at term, those who experienced PTB tended to be older and had a higher prevalence of multiple pregnancy, *in vitro* fertilization, intrahepatic cholestasis of pregnancy, preeclampsia, and pregnancy-induced hypertension. Several laboratory parameters also differed between groups, including higher neutrophil counts and lower levels of hemoglobin, albumin, prealbumin, and retinol-binding protein among women with preterm birth. SMDs indicated moderate to large imbalances for selected predictors, particularly multiple pregnancy, preeclampsia, and markers related to liver function and nutritional status. These comparisons were intended to describe the characteristics of the study population and to provide context for subsequent model development.

### 3.2 Feature selection using LASSO regularization

**Figure 3:**
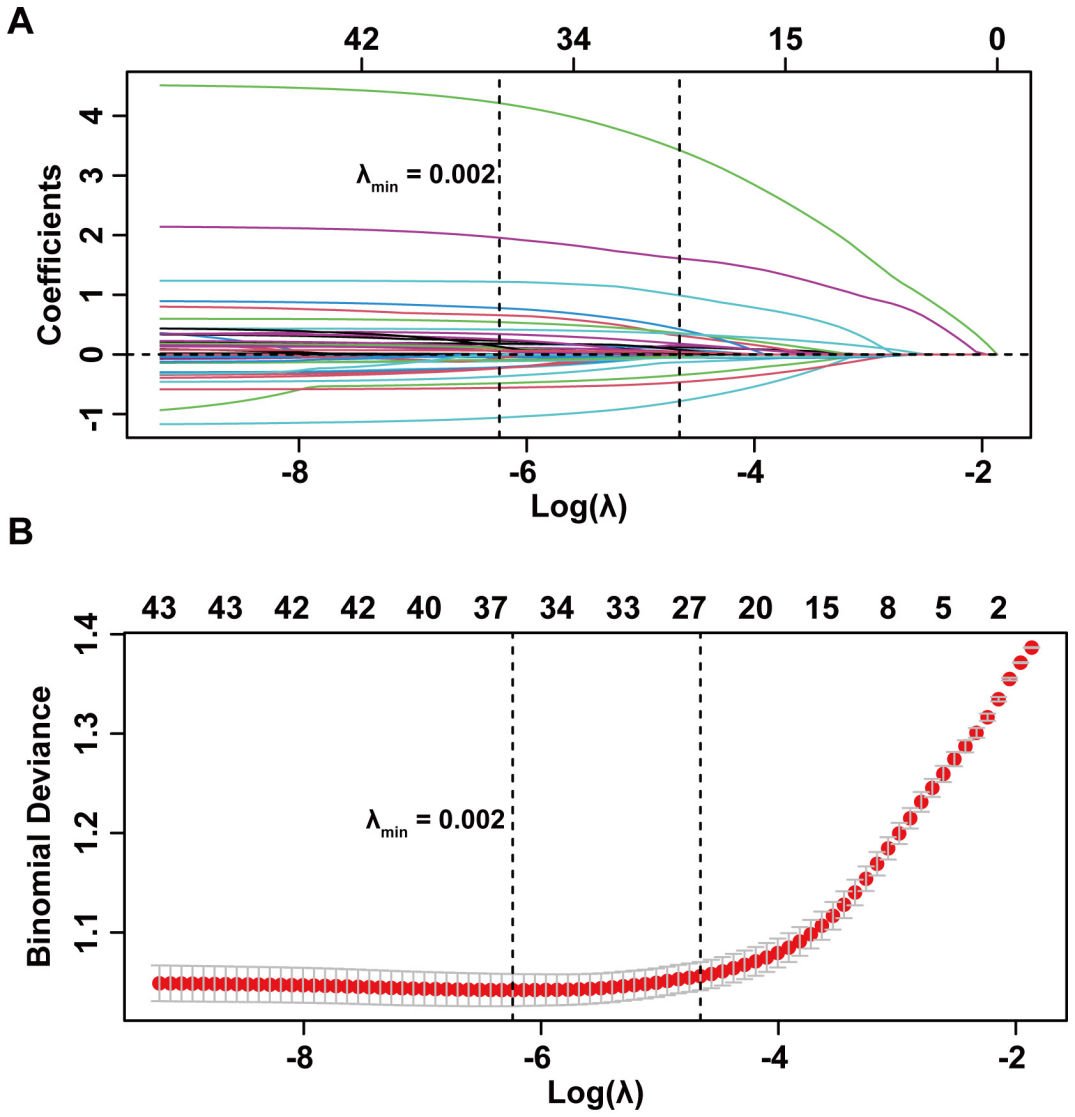
Feature selection using LASSO logistic regression in the derivation cohort. **(A)** LASSO coefficient profiles of the 42 candidate predictors as a function of the log-transformed regularization parameter (log λ). Each curve represents the trajectory of a predictor coefficient with increasing penalization. **(B)** Ten-fold cross-validation curve showing the binomial deviance as a function of log λ. The optimal penalty parameter (λ_min_ = 0.002) was selected at the minimum deviance, yielding 37 predictors with non-zero coefficients for subsequent model development. Numbers above the plots indicate the number of retained predictors at each λ value.

To obtain a parsimonious and reproducible predictor set while mitigating potential overfitting, LASSO logistic regression was applied to the derivation cohort for feature selection. This step was used as a regularization-based predictor screening procedure rather than as a major dimensionality-reduction approach. As shown in Figure 3A, regression coefficients of candidate predictors were progressively shrunk toward zero with increasing penalization. The optimal penalty parameter was determined using 10-fold cross-validation, with the minimum binomial deviance achieved at λ_min_ = 0.002.

At λ_min_, a total of 37 predictors retained non-zero coefficients and were selected for subsequent model development (Figure 3B). These features represented a combination of maternal characteristics, pregnancy-related complications, hematological indices, infection-related variables, and biochemical and metabolic markers. The selected feature set was carried forward for training and comparison of machine learning models in the derivation cohort.

### 3.3 Model development and comparison in the derivation cohort

**Figure 4:**
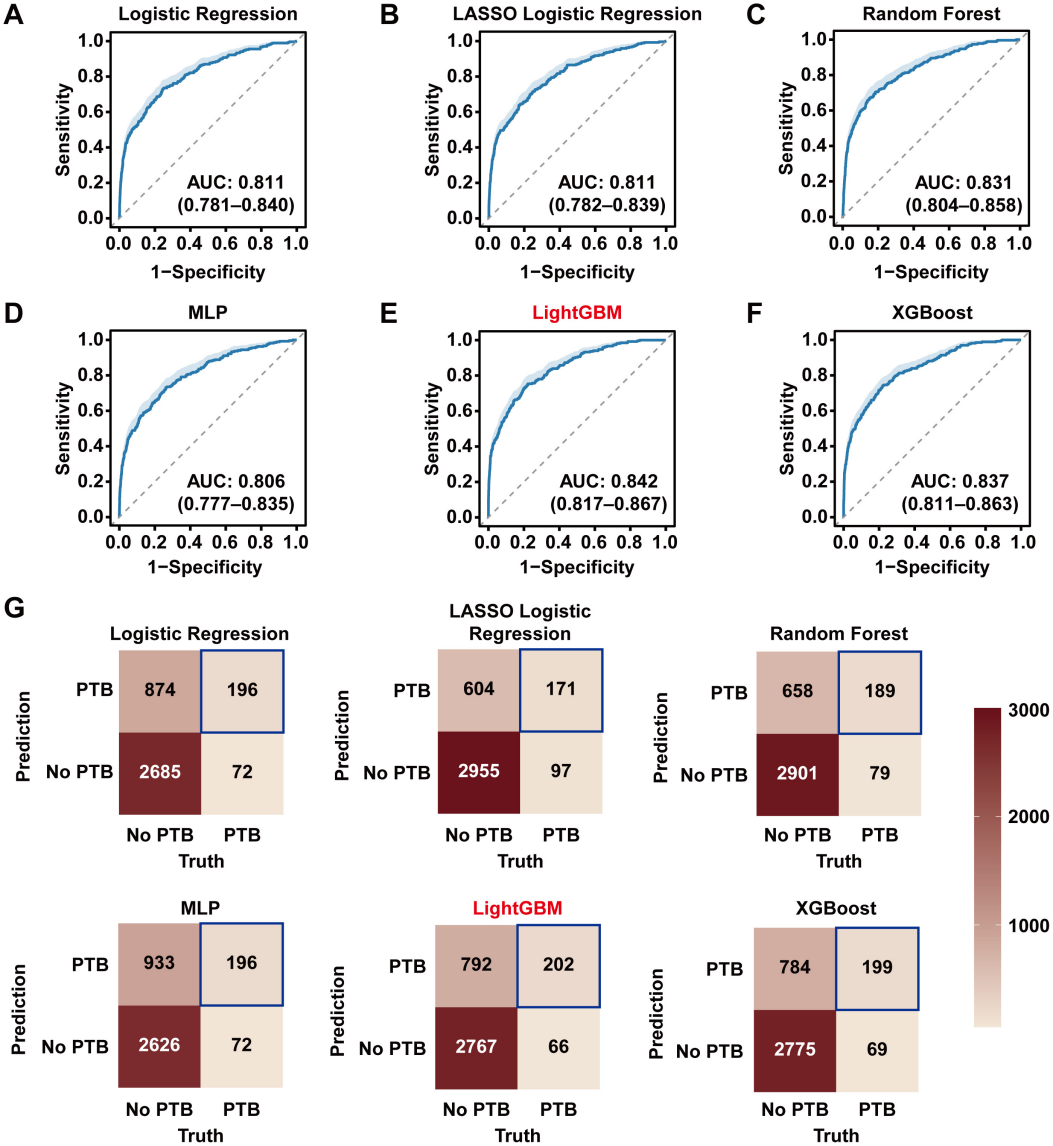
Performance comparison of prediction models in the derivation cohort. **(A–F)** Receiver operating characteristic (ROC) curves for logistic regression, LASSO logistic regression, random forest (RF), multilayer perceptron (MLP), Light gradient boosting machine (LightGBM), and extreme gradient boosting (XGBoost) models, respectively. Shaded areas indicate 95% confidence intervals for the AUC. **(G)** Confusion matrices for each model based on the internal test set of the derivation cohort, illustrating the distribution of predicted versus observed preterm birth (PTB) outcomes.

Using the selected predictors, multiple predictive models were developed and compared in the derivation cohort, including logistic regression with all candidate predictors, LASSO-regularized logistic regression, and four machine learning models (RF, MLP, LightGBM, and XGBoost). Expanded performance metrics in the internal test set are summarized in Table 2, and ROC curves and confusion matrices are shown in Figure 4.

Table 2Expanded performance metrics of candidate models in the internal test set and the final LightGBM model in the prospective validation cohort.

**Table:** 

Cohort	Model	AUC (95% CI)	Sens.	Spec.	PPV	NPV	F1-score	PR-AUC
Panel A. Discrimination and threshold-dependent performance								
Internal test set	Logistic regression	0.811 (0.781–0.840)	0.731	0.754	0.183	0.974	0.293	0.393
Internal test set	LASSO logistic regression	0.811 (0.782–0.839)	0.638	0.830	0.221	0.968	0.328	0.392
Internal test set	Random forest	0.831 (0.804–0.858)	0.705	0.815	0.223	0.973	0.339	0.375
Internal test set	MLP	0.806 (0.777–0.835)	0.731	0.738	0.174	0.973	0.281	0.392
Internal test set	LightGBM	0.842 (0.817–0.867)	0.754	0.777	0.203	0.977	0.320	0.472
Internal test set	XGBoost	0.837 (0.811–0.863)	0.743	0.780	0.202	0.976	0.318	0.463
Prospective validation cohort	LightGBM	0.823 (0.805–0.840)	0.730	0.753	0.225	0.966	0.343	0.496

**Table:** 

Cohort	Model	Calibration intercept	Calibration slope	Brier score
Panel B. Calibration performance				
Internal test set	Logistic regression	−2.530	0.845	0.155
Internal test set	LASSO logistic regression	–2.530	0.847	0.155
Internal test set	Random forest	–0.519	1.270	0.057
Internal test set	MLP	–2.245	0.821	0.127
Internal test set	LightGBM	–2.078	1.129	0.108
Internal test set	XGBoost	–2.241	1.268	0.125
Prospective validation cohort	LightGBM	–1.953	0.999	0.120

The internal test set was the held-out evaluation subset of the derivation cohort. Threshold-dependent metrics were calculated using model-specific thresholds in the internal test set. For the prospective validation cohort, the prespecified LightGBM threshold derived from the internal test set was applied without re-optimizing the threshold in the validation cohort. AUC confidence intervals were estimated using DeLong's method. AUC, area under the receiver operating characteristic curve; CI, confidence interval; Sens., sensitivity; Spec., specificity; PPV, positive predictive value; NPV, negative predictive value; PR-AUC, area under the precision-recall curve; RF, random forest; MLP, multilayer perceptron; LightGBM, light gradient boosting machine; XGBoost, extreme gradient boosting.

Logistic regression using all 42 candidate predictors achieved an AUC of 0.811 (95% CI, 0.781–0.840), whereas LASSO-regularized logistic regression using the selected set of 37 predictors achieved an identical AUC of 0.811 (95% CI, 0.782–0.839). The two logistic models also showed generally similar threshold-dependent performance, although LASSO-regularized logistic regression had higher specificity (0.830 vs. 0.754) and PPV (0.221 vs. 0.183). These findings indicate that LASSO-based predictor screening did not compromise discrimination at the feature-selection stage and supported the use of the selected predictor set for subsequent machine learning model development.

Among the machine learning models, LightGBM showed the highest AUC point estimate in the internal test set, with an AUC of 0.842 (95% CI, 0.817–0.867), followed by XGBoost (AUC = 0.837; 95% CI, 0.811–0.863), RF (AUC = 0.831; 95% CI, 0.804–0.858), and MLP (AUC = 0.806; 95% CI, 0.777–0.835). Pairwise DeLong tests showed that LightGBM had a higher AUC than logistic regression (*P* = 0.002), LASSO-regularized logistic regression (*P* = 0.002), and MLP (*P* = 0.001), whereas its AUC did not differ significantly from RF (*P* = 0.140) or XGBoost (*P* = 0.273). The full pairwise model-comparison results are provided in Supplementary Table S7.

Beyond ROC-AUC, LightGBM showed the highest PR-AUC among the candidate models (0.472), together with relatively high sensitivity (0.754), specificity (0.777), and NPV (0.977). XGBoost showed a similar discrimination profile, with an AUC of 0.837 and PR-AUC of 0.463, whereas RF had the highest F1-score (0.339) and PPV (0.223). Calibration-related metrics and Brier scores varied across models and are summarized in Table 2. LightGBM was selected for subsequent prospective validation and model interpretation based on its overall performance profile, including discrimination, PR-AUC, sensitivity-specificity balance, NPV, and clinical interpretability.

To further assess whether the final machine learning model was sensitive to the LASSO-based feature selection step, we retrained the LightGBM model using all 42 candidate predictors without LASSO-based preselection. In the internal test set, the all-predictor LightGBM model achieved an AUC of 0.847 (95% CI, 0.823–0.871), which was comparable to the AUC of 0.842 (95% CI, 0.817–0.867) achieved by the primary LightGBM model using the 37 LASSO-selected predictors (Supplementary Table S8). These findings indicate that final model performance was not substantially altered when all candidate predictors were used and that the exclusion of five predictors during the LASSO step had limited impact on the final model.

### 3.4 Model performance, clinical utility, and risk stratification in the prospective validation cohort

**Figure 5:**
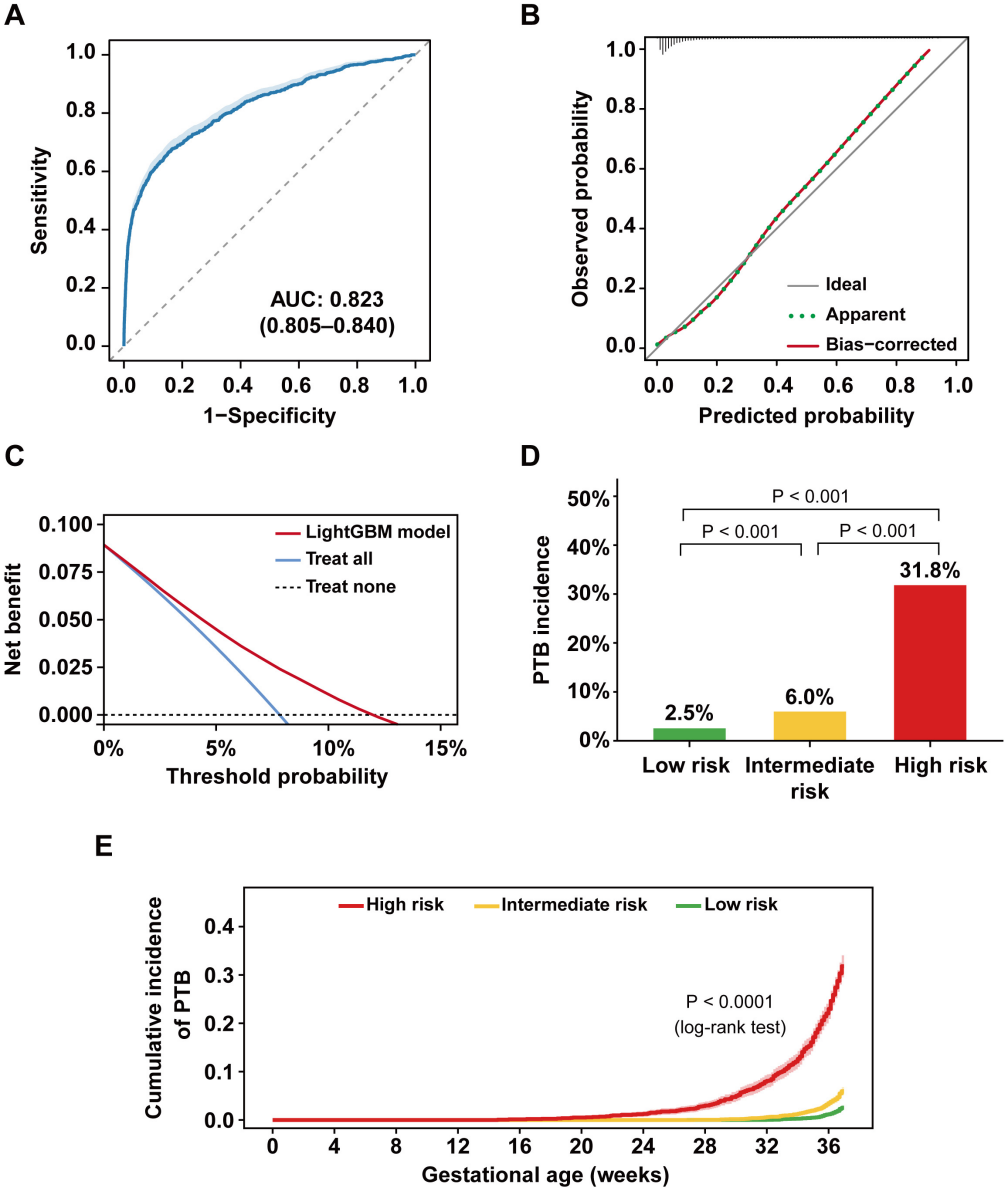
Performance, calibration, clinical utility, and risk stratification of the LightGBM model in the prospective validation cohort. **(A)** Receiver operating characteristic (ROC) curve with the area under the curve (AUC) and 95% confidence interval. **(B)** Calibration plot showing the relationship between predicted probabilities and observed outcomes, including the apparent curve, bootstrap bias-corrected curve, and the ideal reference line. **(C)** Decision curve analysis (DCA) of the LightGBM model in the prospective validation cohort. The model showed higher net benefit than both default strategies within lower threshold probabilities, particularly between 5% and 10%, which are relevant to screening-oriented mid-pregnancy PTB risk assessment. **(D)** Incidence of preterm birth (PTB) across prespecified risk strata (low, intermediate, and high risk) defined by predicted probabilities. **(E)** Cumulative incidence of PTB across risk strata estimated using the Kaplan–Meier method; differences among groups were assessed using the log-rank test.

The final LightGBM model was evaluated in the temporally independent prospective validation cohort. The model maintained good discrimination, with an AUC of 0.823 (95% CI, 0.805–0.840) (Figure 5A). Using the prespecified LightGBM threshold derived from the internal test set, the model achieved a sensitivity of 0.730, specificity of 0.753, PPV of 0.225, NPV of 0.966, and F1-score of 0.343 in the prospective validation cohort (Table 2). The PR-AUC was 0.496, indicating that the model retained useful ranking performance for PTB events despite the relatively low event rate. The calibration slope was 0.999, with a Brier score of 0.120, and the bootstrap bias-corrected calibration curve showed general agreement between predicted and observed risks across the range of predicted probabilities (Figure 5B).

Decision curve analysis further supported the potential clinical utility of the LightGBM model, particularly within the lower threshold probabilities relevant to screening-oriented mid-pregnancy PTB risk assessment (Figure 5C). At threshold probabilities of 5% and 10%, the model achieved net benefit values of 0.044 and 0.007, respectively, exceeding both default strategies of classifying all women as high risk or none as high risk. Compared with classifying all women as high risk, model-guided risk assessment corresponded to net reductions of 4.4 and 16.9 unnecessary intensified-surveillance interventions per 100 women at the 5% and 10% thresholds, respectively. At the higher reference threshold of 15%, the model remained more favorable than classifying all women as high risk, corresponding to a net reduction of 28.6 unnecessary interventions per 100 women; however, its net benefit did not exceed the classify-none strategy. These findings indicate that the model's clinical utility is most relevant within lower threshold probabilities where closer surveillance, referral, or individualized counseling may be considered. Detailed net benefit values and intervention trade-offs are provided in Supplementary Table S9.

Using the prespecified risk stratification strategy, women classified as high risk had a markedly higher incidence of PTB than those in the intermediate- and low-risk groups (31.8% vs. 6.0% and 2.5%, respectively; *P* < 0.001 for all pairwise comparisons) (Figure 5D). Compared with the low-risk group, the high-risk group had a 12.59-fold higher observed PTB risk (95% CI, 10.33–15.34). Consistent with these findings, time-to-event analysis showed sustained separation of cumulative PTB incidence across risk groups throughout gestation (log-rank test, *P* < 0.0001) (Figure 5E). Notably, the cumulative incidence curve for the high-risk group diverged earlier and rose more steeply, suggesting earlier occurrence and faster accumulation of PTB events compared with the intermediate- and low-risk groups, whereas the low-risk group maintained consistently low cumulative incidence across gestation.

Sensitivity analyses using alternative derivation cohort–based percentile thresholds demonstrated consistent risk gradients across low-, intermediate-, and high-risk groups (Supplementary Table S10). When the high-risk threshold was varied from the top 10% to the top 20% while the low-risk threshold was fixed at the bottom 50%, the observed PTB incidence in the high-risk group ranged from 26.2% to 39.4%, compared with 2.5% in the low-risk group. The corresponding high vs. low risk ratios ranged from 10.35 to 15.60, and all trend tests were significant (*P* for trend < 0.001). The top 10% threshold defined a smaller and more risk-enriched high-risk subgroup, but it captured fewer PTB events; conversely, the top 20% threshold captured more PTB events, but at the cost of a larger and less risk-enriched high-risk subgroup.

Similarly, when the low-risk threshold was varied from the bottom 40% to the bottom 60% while the high-risk threshold was fixed at the top 15%, the observed PTB incidence in the low-risk group remained consistently low, ranging from 2.4% to 2.9%. Compared with the bottom 40% threshold, the primary bottom 50% threshold identified a larger low-risk subgroup while maintaining a similarly low observed PTB incidence (2.5%). Although the bottom 60% threshold further expanded the low-risk subgroup, it was accompanied by a modest increase in observed PTB incidence to 2.9%. Collectively, these findings further support the clinical rationale for the primary top 15% and bottom 50% thresholds, indicating that this strategy provides a pragmatic balance among high-risk enrichment, PTB event capture, low-risk group size, and clinical manageability.

### 3.5 Global feature importance and contribution to model predictions

To improve interpretability of the LightGBM model, SHapley Additive exPlanations (SHAP) analysis was used to quantify the contribution of individual predictors to model output at the population level. Global feature importance based on mean absolute SHAP values for all selected predictors is summarized in Supplementary Table S11, providing a comprehensive ranking of the 37 features retained after LASSO-based selection. Among these, inflammatory markers, liver-related enzymes, and maternal characteristics consistently ranked highest.

**Figure 6:**
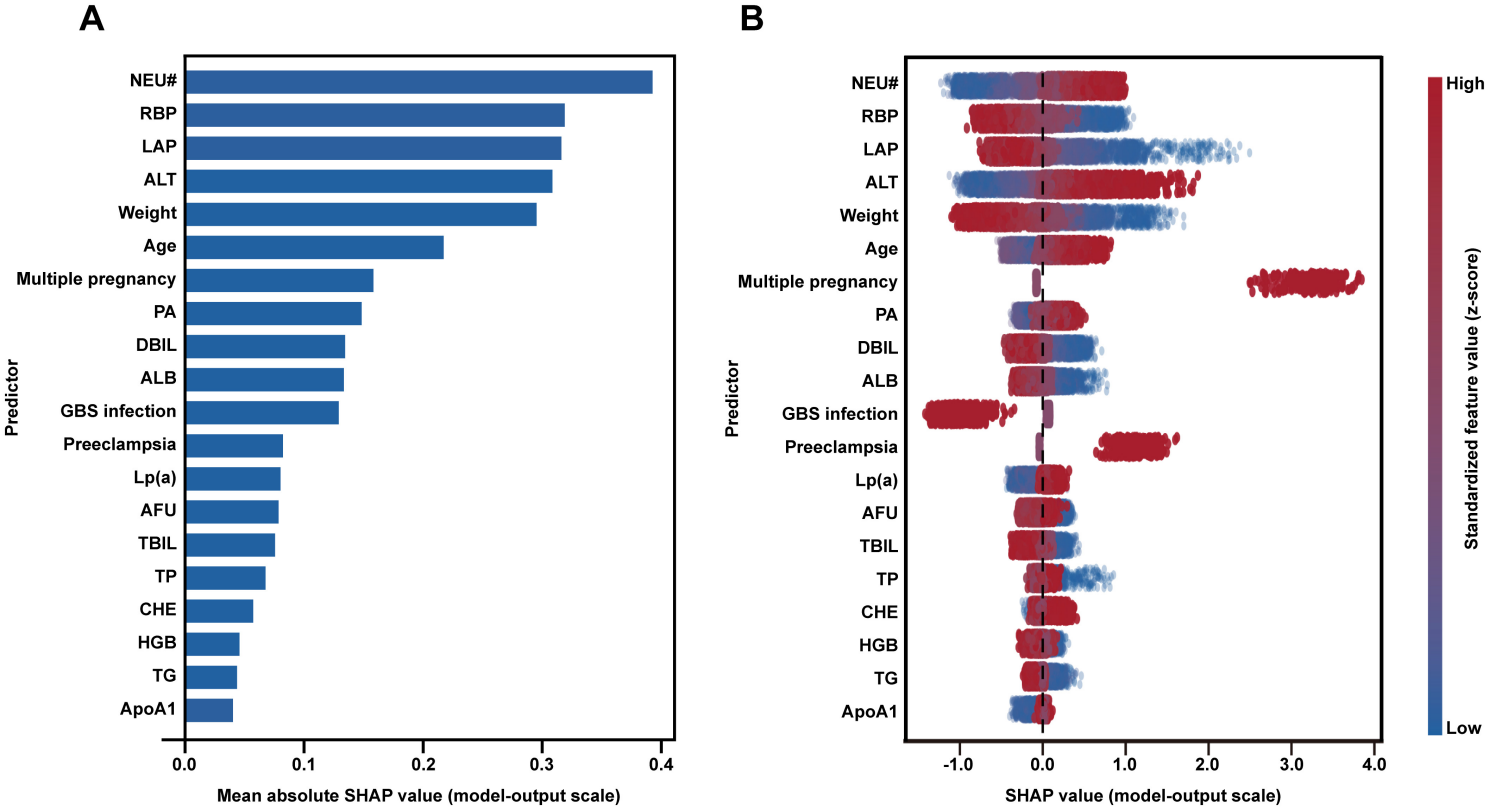
Global feature importance and SHAP-based interpretation of the LightGBM model for preterm birth prediction. **(A)** Global feature importance ranked by mean absolute SHAP values, reflecting the overall contribution of each predictor to the model output across the study population. **(B)** SHAP summary plot illustrating the distribution of SHAP values for the top-ranked features. Each point represents an individual participant, with color indicating the standardized feature value (red = high, blue = low). Positive SHAP values indicate contributions toward a higher model-predicted risk of preterm birth, whereas negative SHAP values indicate contributions toward a lower predicted risk. SHAP, SHapley Additive exPlanations; NEU#, neutrophil count; RBP, retinol-binding protein; LAP, leucine aminopeptidase; ALT, alanine aminotransferase; PA, prealbumin; DBIL, direct bilirubin; ALB, albumin; GBS, Group B Streptococcus; Lp(a), lipoprotein (a); AFU, α-L-fucosidase; TBIL, total bilirubin; TP, total protein; CHE, cholinesterase; HGB, hemoglobin; TG, triglycerides; ApoA1, apolipoprotein A1.

For clarity and visualization, the top 20 features ranked by mean absolute SHAP value are displayed in Figure 6. Neutrophil count (NEU#) emerged as the most influential predictor, followed by retinol-binding protein (RBP), leucine aminopeptidase (LAP), alanine aminotransferase (ALT), maternal weight, and maternal age (Figure 6A). Several pregnancy-related clinical conditions, including multiple pregnancy, group B Streptococcus (GBS) infection, and preeclampsia, also showed substantial contributions. The SHAP summary plot further illustrated both the direction and magnitude of feature effects across the study population (Figure 6B). Higher NEU#, ALT, maternal age, multiple pregnancy, and preeclampsia were associated with positive SHAP values, indicating increased predicted risk of PTB. In contrast, higher levels of RBP, LAP, maternal weight, direct bilirubin (DBIL), and albumin (ALB) were predominantly associated with negative SHAP values, corresponding to lower predicted risk.

### 3.6 Nonlinear and threshold effects revealed by SHAP dependence analysis

**Figure 7:**
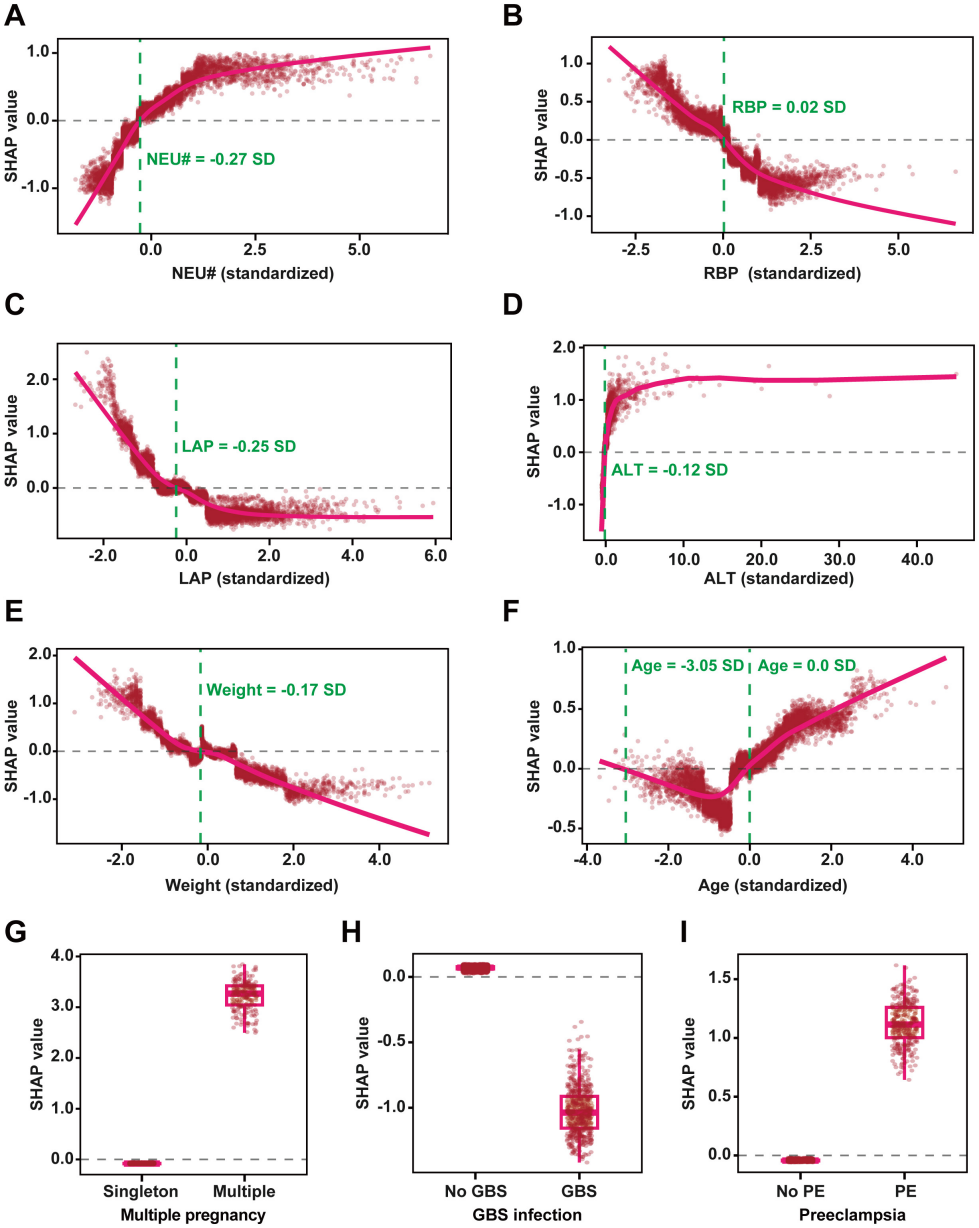
SHAP dependence analysis of key predictors in the LightGBM model. **(A–F)** SHAP dependence plots for representative continuous variables, including neutrophil count (NEU#), retinol-binding protein (RBP), leucine aminopeptidase (LAP), alanine aminotransferase (ALT), maternal weight, and maternal age. Each point represents an individual observation, and the solid curve represents a locally weighted smoothing (LOWESS) fit to visualize the overall trend of SHAP values across the standardized feature range. **(G–I)** SHAP value distributions for selected categorical variables, including multiple pregnancy, Group B Streptococcus (GBS) infection, and preeclampsia (PE). Positive SHAP values indicate contributions toward higher model-predicted risk of preterm birth, whereas negative SHAP values indicate contributions toward lower predicted risk.

To further elucidate how key predictors influenced model predictions, SHAP dependence plots were generated for representative continuous and categorical variables among the top-ranked features (Figure 7). These plots revealed both nonlinear effects and threshold-dependent relationships between predictors and the predicted risk of PTB.

As NEU# increased from relatively low values toward the mid-range of its distribution, SHAP values rose sharply, indicating an increasing contribution to the model-predicted PTB risk. At higher NEU# levels, SHAP values tended to plateau, suggesting diminishing marginal contributions of further increases to the model output (Figure 7A). In contrast, RBP and LAP showed predominantly inverse patterns, with higher standardized values generally corresponding to lower SHAP values across most of their distributions, indicating reduced contributions to the model-predicted risk (Figures 7B, C). ALT exhibited a non-linear pattern with a rapid rise in SHAP values at lower levels followed by an apparent saturation at higher levels, suggesting that increases in ALT within the lower range contributed substantially to the model output, whereas additional increases at higher levels added relatively limited incremental contribution (Figure 7D). Maternal weight was associated with largely negative SHAP values across most of the observed range, with higher weight corresponding to lower contributions to the model-predicted risk (Figure 7E). Maternal age demonstrated a non-linear, U-shaped pattern, where both lower and higher standardized age values were associated with positive SHAP values, while intermediate values were closer to or below zero, indicating greater model-attributed risk contributions at the extremes of maternal age (Figure 7F).

For categorical predictors, multiple pregnancy was consistently associated with high positive SHAP values compared with singleton pregnancy, reflecting a strong and stable contribution to elevated model-predicted PTB risk (Figure 7G). GBS infection did not show a pronounced positive shift in SHAP values; values were centered around or below zero, suggesting a limited contribution to increasing model-predicted PTB risk in this cohort (Figure 7H). In contrast, preeclampsia was associated with markedly positive SHAP values, indicating a substantial contribution to higher model-predicted risk among affected women (Figure 7I). Collectively, these SHAP dependence analyses illustrate feature-specific and non-linear relationships between clinical variables and model predictions, providing transparent insight into the decision patterns of the LightGBM model.

## 4 Discussion

In this study, we developed and prospectively validated a screening-oriented machine learning model for mid-pregnancy prediction of PTB using routinely available maternal characteristics and laboratory indicators. The model was designed for women receiving routine antenatal care between 14 and 27 completed gestational weeks in a tertiary maternity center, including both singleton and multiple pregnancies, rather than for a preselected high-risk obstetric population. Accordingly, its intended use is best framed as mid-pregnancy risk stratification in a tertiary antenatal care setting, not as a universal prediction model for all pregnant populations or healthcare systems.

Previous efforts to predict PTB have largely relied on traditional regression models or isolated clinical risk factors, such as obstetric history, cervical length, or biochemical markers, with limited and often inconsistent predictive performance (15–17). More recent studies have explored machine learning–based approaches for PTB prediction, yet several methodological and translational challenges remain. A systematic review of machine learning–based PTB prediction models reported substantial limitations in reporting and methodological quality, including concerns related to risk of bias, sample size, predictor selection, and incomplete calibration assessment (18). Liang et al. (11) reported a protein-based machine learning model using LC–MS/MS profiling, which demonstrated promising discrimination; however, the requirement for advanced proteomic platforms and the absence of prospective validation indicate that further evaluation is needed before large-scale clinical implementation. Zhou et al. (12) incorporated prospective validation in a transformer-based model by integrating multi-omics information derived from cell-free DNA and cell-free RNA sequencing, but the reliance on sequencing-based assays and specialized infrastructure may limit scalability in routine clinical practice. Other studies have focused on readily available clinical variables but were restricted to retrospective designs without prospective validation, raising concerns regarding generalizability (10, 19, 20). Moreover, several reported models achieved only modest discriminative performance, with AUC values frequently below 0.80, potentially constraining their utility for effective risk stratification (10, 21, 22). In contrast, the present model was based entirely on routinely collected clinical and laboratory indicators spanning multiple physiological domains. This design was intended to balance predictive performance with practical feasibility, because all predictors can be obtained during standard mid-pregnancy antenatal assessment without additional omics-based or specialized assays.

In the temporally independent prospective validation cohort from a subsequent time period within the same tertiary maternity center, the LightGBM model maintained good discrimination (AUC = 0.823), useful precision-recall performance (PR-AUC = 0.496), and acceptable probabilistic performance, with a calibration slope of 0.999 and a Brier score of 0.120. This multidimensional evaluation is consistent with recent reporting and appraisal guidance for AI-based clinical prediction models, which emphasizes that discrimination, calibration, overall prediction error, and clinical utility should be considered together rather than relying on AUC alone (23). Beyond discrimination alone, decision curve analysis suggested potential clinical value within lower threshold probabilities relevant to screening-oriented mid-pregnancy risk assessment, particularly between 5% and 10%. At these thresholds, model-guided assessment provided greater net benefit than default strategies and corresponded to fewer unnecessary intensified-surveillance interventions compared with classifying all women as high risk. These findings suggest that the model may be most useful in clinical scenarios where the downstream response involves relatively low-burden actions, such as closer surveillance, referral, or individualized counseling, rather than immediate therapeutic intervention.

The clinical relevance of the model also lies in its ability to translate routinely available mid-pregnancy data into actionable risk stratification. In a practical workflow, the model could be embedded into the antenatal information system once routine clinical and laboratory data are available, generating both an individualized predicted probability and a prespecified risk category during mid-pregnancy visits. A high-risk result should be interpreted as a prompt for clinician review and risk-informed care planning rather than as an automatic trigger for treatment. Potential downstream actions may include closer antenatal surveillance, reassessment of modifiable risk factors, cervical length assessment when clinically appropriate, referral to a maternal–fetal medicine specialist, and individualized counseling regarding warning symptoms and follow-up. Conversely, a low-risk result should not replace standard antenatal care, but may help avoid unnecessary escalation of monitoring in women with consistently low predicted risk.

Using probability thresholds derived from the derivation cohort and fixed before application to the prospective validation cohort, the model identified a high-risk subgroup with substantially elevated observed PTB incidence and a large low-risk subgroup with consistently low observed incidence. Women classified as high risk not only had a higher overall incidence of PTB, but also showed earlier accumulation of PTB events across gestation. This temporal separation is clinically relevant because earlier-onset PTB is associated with disproportionately greater neonatal morbidity and mortality (24, 25), and it supports the potential use of model-based risk stratification for anticipatory surveillance. Sensitivity analyses using alternative derivation cohort–based thresholds further supported the clinical rationale for the primary top 15% and bottom 50% strategy, indicating that this approach provides a pragmatic balance among high-risk enrichment, PTB event capture, low-risk group size, and clinical manageability. Collectively, these findings suggest that the model may serve as a decision-support aid for mid-pregnancy screening, complementing clinical judgment and helping prioritize antenatal resources according to predicted PTB risk.

A major barrier to the clinical adoption of machine learning models is the perceived opacity of complex algorithms, often described as the “black-box” problem (26). To improve transparency, we used SHAP-based interpretability analyses to characterize how individual variables contributed to the LightGBM model output at both the population and feature-dependence levels. These analyses should be interpreted as explanations of model behavior rather than evidence of independent associations or causal mechanisms. Accordingly, the SHAP findings are best viewed as hypothesis-generating signals that may help clinicians understand which routinely available clinical and laboratory features were used by the model to assign higher or lower predicted PTB risk.

Within this interpretive framework, several high-ranking features were clinically plausible and broadly aligned with existing knowledge of PTB risk. Neutrophil count showed the strongest contribution to model-predicted risk, suggesting that inflammatory information contained in routine blood tests was important for model discrimination. This pattern is consistent with prior evidence linking maternal inflammatory activation to PTB-related processes (27). The nonlinear SHAP dependence pattern, characterized by a rapid increase in predicted risk from lower to moderate neutrophil levels followed by attenuation at higher values, further suggests that the model captured a nonlinear contribution of neutrophil count to predicted risk rather than a simple linear dose–response pattern.

Similarly, liver-related biochemical markers and nutritional indicators, including ALT, ALB, RBP, LAP, and maternal weight, contributed to model predictions in clinically interpretable directions. The positive model-attributed contribution of ALT was consistent with previous evidence linking maternal liver dysfunction and metabolic liver disease with adverse pregnancy outcomes, including PTB (28, 29). Conversely, higher ALB, RBP, LAP, and maternal weight were generally associated with lower model-predicted risk, a pattern broadly compatible with epidemiological evidence relating maternal nutritional status and energy reserves to birth outcomes (30, 31). These findings suggest that the model may have captured risk information related to maternal hepatic, metabolic, and nutritional status.

The SHAP dependence plots further illustrated that several predictors contributed to model output in nonlinear ways. Maternal age showed a U-shaped model-attributed contribution, broadly consistent with large-scale epidemiological evidence that both younger and advanced maternal ages are associated with higher PTB risk (32). Multiple pregnancy and preeclampsia showed consistently positive contributions to predicted risk, corresponding to well-recognized risk-related obstetric profiles (33, 34). These findings support the clinical face validity of the model because the model used information from variables that are already recognized as clinically relevant to PTB risk. In contrast, GBS colonization did not show a strong positive contribution to predicted risk in this cohort, which may reflect context-specific effects related to screening and treatment practices. Overall, the SHAP analyses increased the transparency of the LightGBM model by describing how predictors influenced model output, but these model-derived signals should be regarded as hypothesis-generating and require further validation in studies designed to evaluate biological pathways or modifiable clinical targets.

Several limitations merit consideration. First, although the study included a large sample size and a prospective validation cohort, both model development and validation were conducted within the same tertiary maternity center. Therefore, the validation should be interpreted as temporal prospective validation within a tertiary-care setting rather than multicenter external validation. The model's transportability to community hospitals, primary antenatal care settings, geographically diverse populations, or healthcare systems with different referral patterns remains to be established. Second, PTB is a heterogeneous outcome that includes both spontaneous and medically indicated deliveries, which may differ in etiology and predictors. Because the original dataset did not contain a reliable and consistently structured field distinguishing these subtypes, we did not perform subtype-specific performance analyses to avoid potential misclassification bias. Accordingly, the model should be interpreted as predicting overall PTB risk rather than subtype-specific PTB risk or subtype-specific causal pathways. Predictors identified by the model may reflect shared risk information across multiple pathways leading to PTB, including inflammatory, placental, metabolic, hypertensive, and fetal indications. Future studies with standardized and granular PTB phenotyping are needed to evaluate whether separate models for spontaneous and medically indicated PTB could improve prediction performance and clinical interpretability. Third, although the model was developed using routinely available clinical and laboratory variables and may therefore be feasible for integration into antenatal care, clinical implementation was not directly evaluated in this study. The present analysis did not test how the model would perform when embedded into real-time antenatal workflows, how clinicians would respond to a high-risk prediction, or which specific combination of follow-up actions would provide the greatest benefit. Potential actions after a high-risk result may include closer antenatal surveillance, reassessment of modifiable risk factors, cervical length assessment when clinically appropriate, referral to maternal–fetal medicine specialists, and individualized counseling; however, whether such model-guided management would improve maternal or neonatal outcomes remains unknown. Future studies should therefore evaluate workflow integration, clinician adherence, patient acceptability, health-economic impact, and, most importantly, whether model-guided surveillance or intervention strategies improve clinical outcomes beyond standard care.

## 5 Conclusion

In summary, this study developed and prospectively validated a machine learning–based model for mid-pregnancy prediction of PTB using routinely collected clinical and laboratory data. By integrating multidimensional yet readily available predictors, the LightGBM model demonstrated stable discrimination, good calibration, clinical net benefit, and meaningful risk stratification in a temporally independent prospective validation cohort within the same tertiary maternity care system. The model is positioned as a screening-oriented decision-support tool rather than a definitive diagnostic instrument, with the potential to support earlier identification of women at increased overall PTB risk and to inform more targeted antenatal surveillance during mid-pregnancy. In a potential implementation pathway, the model could be embedded into electronic antenatal care systems after routine mid-pregnancy clinical and laboratory data become available, generating risk estimates and predefined risk categories for clinician review and risk-informed follow-up planning. Further multicenter external validation, standardized PTB subtype phenotyping, dynamic risk updating across gestation, and prospective evaluation of model-guided clinical pathways are needed before broader implementation.

## Data Availability

The original contributions presented in the study are included in the article/Supplementary material, further inquiries can be directed to the corresponding author.
