# Supplementary material for: A machine learning-based risk prediction model for early preterm birth: development and prospective validation
Source: Front Med (Lausanne). 2026 Jul 22;13:1888022. doi: 10.3389/fmed.2026.1888022 (PMC13438318; doi:10.3389/fmed.2026.1888022)
Supplement: Supplementary file 1 [file Supplementary_file_1.docx]

Supplementary Material

# 1 Supplementary Tables

**Table S1.** Timing and ascertainment rules for predictors in relation to the prediction landmark

| **Predictor domain** | **Predictors included** | **Data source** | **Timing rule** | **Handling of post-landmark information** | **Missingness handling** |
| --- | --- | --- | --- | --- | --- |
| Maternal demographic characteristics | Maternal age, maternal weight, obesity | Antenatal electronic medical records | Values recorded before or at the individual prediction landmark were used. | Information updated after the prediction landmark was not used. | Missing continuous variables were imputed using median values derived from the training set. |
| Obstetric history | Gravidity, parity, mode of conception, multiple pregnancy, in vitro fertilization | Antenatal electronic medical records | Information documented before or at the prediction landmark was used. | Changes or records added after the prediction landmark were not used for predictor definition. | Categorical variables had no missing values; otherwise coded according to the prespecified data dictionary. |
| Pre-existing or baseline maternal conditions | Uterine fibroid, cardiac arrhythmia, thyroid disease, hepatitis B virus infection | Antenatal records and diagnosis records | Diagnoses documented before or at the prediction landmark were coded as present. | Diagnoses first recorded after the prediction landmark were coded as absent at the time of prediction. | Categorical variables had no missing values; otherwise coded as absent if no eligible diagnosis record was available before or at the landmark. |
| Pregnancy-related complications | Gestational diabetes mellitus, intrahepatic cholestasis of pregnancy, preeclampsia, pregnancy-induced hypertension | Antenatal diagnosis records, laboratory records, and obstetric assessment records | The first documented diagnosis date was compared with the individual prediction landmark. A complication was coded as present only if the diagnosis was documented on or before the landmark. | Diagnoses first recorded after the landmark were not used as predictors and were coded as absent at the time of prediction. | Coded as absent if no eligible diagnosis record was available before or at the landmark. |
| Infection-related variables | Group B Streptococcus infection, vaginitis | Microbiology reports, laboratory records, and antenatal diagnosis records | Test results or diagnoses available before or at the prediction landmark were used. | Test results or diagnoses first available after the landmark were excluded from predictor definition. | Coded as absent if no eligible positive result or diagnosis was available before or at the landmark. |
| Hematological indices | Hemoglobin, neutrophil count, lymphocyte count | Laboratory information system | The most recent eligible result within 14–27 completed gestational weeks and on or before the prediction landmark was used. | Laboratory results obtained after the landmark were excluded. | Missing values were imputed using median values derived from the training set. |
| Biochemical, metabolic, and liver-related markers | Retinol-binding protein, prealbumin, alpha-L-fucosidase, leucine aminopeptidase, cholinesterase, total bilirubin, direct bilirubin, total protein, albumin, alanine aminotransferase, total cholesterol, triglycerides, low-density lipoprotein cholesterol, apolipoprotein A1, apolipoprotein B, lipoprotein(a) | Laboratory information system | The most recent eligible result within 14–27 completed gestational weeks and on or before the prediction landmark was used. | Laboratory results obtained after the landmark were excluded. | Missing values were imputed using median values derived from the training set. |
| Outcome | Preterm birth status and gestational age at delivery | Delivery records | Outcome was ascertained after the prediction landmark from delivery records. | Outcome information was not used during predictor definition, preprocessing, feature selection, or model development. | Not applicable. |

**Table S2.** Temporal verification of pregnancy-related complications included as predictors

| **Predictor** | **Cohort/subset** | **Women coded positive in model, n** | **First diagnosis on/before prediction landmark, n (%)** | **First diagnosis after prediction landmark, n** | **Handling of post-landmark diagnoses** |
| --- | --- | --- | --- | --- | --- |
| Gestational diabetes mellitus | Training set | 1809 | 100.0 | 0 | Coded as absent at prediction |
| Gestational diabetes mellitus | Internal test set | 785 | 100.0 | 0 | Coded as absent at prediction |
| Gestational diabetes mellitus | Prospective validation cohort | 1973 | 100.0 | 0 | Coded as absent at prediction |
| Intrahepatic cholestasis of pregnancy | Training set | 144 | 100.0 | 0 | Coded as absent at prediction |
| Intrahepatic cholestasis of pregnancy | Internal test set | 53 | 100.0 | 0 | Coded as absent at prediction |
| Intrahepatic cholestasis of pregnancy | Prospective validation cohort | 172 | 100.0 | 0 | Coded as absent at prediction |
| Preeclampsia | Training set | 326 | 100.0) | 0 | Coded as absent at prediction |
| Preeclampsia | Internal test set | 132 | 100.0 | 0 | Coded as absent at prediction |
| Preeclampsia | Prospective validation cohort | 347 | 100.0 | 0 | Coded as absent at prediction |
| Pregnancy-induced hypertension | Training set | 302 | 100.0 | 0 | Coded as absent at prediction |
| Pregnancy-induced hypertension | Internal test set | 141 | 100.0 | 0 | Coded as absent at prediction |
| Pregnancy-induced hypertension | Prospective validation cohort | 325 | 100.0 | 0 | Coded as absent at prediction |

**Table S3.** Missingness distribution across candidate predictors before imputation

| **Predictor** | **Missingness, %** |
| --- | --- |
| Lp(a) | 49.08 |
| TC | 49.08 |
| TG | 49.08 |
| HDL | 49.08 |
| LDL | 49.08 |
| ApoA1 | 49.08 |
| ApoB | 49.08 |
| CRP | 7.01 |
| Weight | 1.49 |
| BMI | 1.49 |
| Hemoglobin | 0.93 |
| Neutrophil count | 0.93 |
| Lymphocyte count | 0.93 |
| RBP | 0.66 |
| Prealbumin | 0.66 |
| AFU | 0.66 |
| LAP | 0.66 |
| Cholinesterase | 0.66 |
| Total bilirubin | 0.60 |
| Direct bilirubin | 0.60 |
| Total protein | 0.60 |
| Albumin | 0.60 |
| ALT | 0.55 |
| AST | 0.55 |
| Height | 0.09 |
| Maternal age | 0.01 |
| Gravidity | 0 |
| Parity | 0 |
| Multiple pregnancy | 0 |
| Gestational diabetes mellitus | 0 |
| Intrahepatic cholestasis of pregnancy | 0 |
| Preeclampsia | 0 |
| Pregnancy-induced hypertension | 0 |
| Thyroid disease | 0 |
| Uterine scar | 0 |
| Obesity | 0 |
| Group B Streptococcus colonization | 0 |
| Vaginitis | 0 |
| Uterine fibroid | 0 |
| Arrhythmia | 0 |
| In vitro fertilization | 0 |
| Hepatitis B virus infection | 0 |

**Table S4.** Hyperparameter search spaces and final selected parameters of candidate machine learning models

| **Model** | **Hyperparameter** | **Search range evaluated** | **Final selected value** |
| --- | --- | --- | --- |
| Random forest | mtry | 5–38 | 8 |
| Random forest | splitrule | gini, extratrees | gini |
| Random forest | min.node.size | 3–20 | 9 |
| Random forest | Random-search iterations | 30 | 30 |
| Random forest | Cross-validation | 5-fold | 5-fold |
| Random forest | Optimization metric | ROC/AUC | ROC/AUC |
| MLP | size | 1–20 | 18 |
| MLP | decay | 2.31 × 10⁻⁵–8.0411 | 8.0411 |
| MLP | maxit | fixed | 800 |
| MLP | MaxNWts | fixed | 20000 |
| MLP | Random-search iterations | 50 | 50 |
| MLP | Cross-validation | 5-fold | 5-fold |
| MLP | Optimization metric | ROC/AUC | ROC/AUC |
| LightGBM | learning_rate | 0.0128–0.1199 | 0.0806 |
| LightGBM | num_leaves | 15–127 | 127 |
| LightGBM | max_depth | -1–10 | 4 |
| LightGBM | min_data_in_leaf | 10–110 | 45 |
| LightGBM | feature_fraction | 0.6026–0.9784 | 0.6026 |
| LightGBM | bagging_fraction | 0.6087–0.9974 | 0.9541 |
| LightGBM | bagging_freq | 0–10 | 10 |
| LightGBM | lambda_l1 | 0.2929–4.5754 | 2.8206 |
| LightGBM | lambda_l2 | 0.0545–9.9780 | 6.0202 |
| LightGBM | scale_pos_weight | fixed | 13.2245 |
| LightGBM | Maximum boosting rounds | fixed | 5000 |
| LightGBM | Optimal boosting iterations | determined by CV | 159 |
| LightGBM | Random-search iterations | 30 | 30 |
| LightGBM | Cross-validation | 5-fold | 5-fold |
| LightGBM | Optimization metric | AUC | AUC |
| XGBoost | eta | 0.0110–0.1995 | 0.0667 |
| XGBoost | max_depth | 3–10 | 3 |
| XGBoost | min_child_weight | 1.0514–9.9254 | 9.9254 |
| XGBoost | subsample | 0.6065–0.9978 | 0.7963 |
| XGBoost | colsample_bytree | 0.6164–0.9975 | 0.6609 |
| XGBoost | gamma | 0.0095–4.9913 | 3.1190 |
| XGBoost | lambda | 0.5669–14.9144 | 14.8495 |
| XGBoost | alpha | 0.0099–4.9501 | 2.0358 |
| XGBoost | scale_pos_weight | fixed | 13.2245 |
| XGBoost | Optimal boosting iterations | determined by CV/early stopping | 157 |
| XGBoost | Random-search iterations | 80 | 80 |
| XGBoost | Cross-validation | 5-fold | 5-fold |
| XGBoost | Optimization metric | AUC | AUC |

**Table S5.** Software environment, SHAP calculation, and random seeds

| **Category** | **Item** | **Version / method / value** | **Role in the analysis** |
| --- | --- | --- | --- |
| Statistical environment | R | 4.5.1 | Main statistical computing environment |
| Model training | caret | 7.0.1 | Model training framework and cross-validation |
| Feature selection | glmnet | 4.1.10 | LASSO-regularized logistic regression |
| Random forest | ranger | 0.18.0 | Random forest model training |
| Gradient boosting | lightgbm | 4.6.0 | LightGBM model training and SHAP calculation |
| Gradient boosting | xgboost | 3.2.1.1 | XGBoost model training |
| Neural network | nnet | 7.3.20 | Multilayer perceptron model training |
| Model evaluation | pROC | 1.19.0.1 | ROC analysis, AUC estimation, and confidence intervals |
| Data processing | dplyr | 1.2.0 | Data manipulation |
| Visualization | ggplot2 | 4.0.2 | Figure generation |
| Matrix processing | Matrix | 1.7.4 | Matrix operations for model input |
| Data processing | data.table | 1.18.2.1 | Data handling and preprocessing |
| Random seed | Model development and random-search tuning | 123 | Data processing, model tuning, and model training reproducibility |
| Random seed | SHAP visualization | 2025 and 123 | Reproducible SHAP visualization and bootstrap/plotting procedures |
| SHAP calculation | Native LightGBM feature-contribution output | predict(..., type = "contrib") | Calculation of SHAP values for the final LightGBM model |
| SHAP post-processing | Bias term removal | Last column removed before feature-level summaries | Exclusion of the model bias term from feature-level SHAP summaries |
| SHAP summary | Global feature importance | Mean absolute SHAP value across individuals | Ranking of predictor contributions |

**Table S6.** Baseline characteristics of pregnant women with and without preterm birth in the derivation cohort

| **Variable** | **Non-PTB**  *n* = 11,864 | **PTB**  *n* = 896 | **SMD** |
| --- | --- | --- | --- |
| Maternal age, years | 29.7 ± 4.1 | 30.6 ± 4.6 | 0.216 |
| Height, cm | 162.0 (158.0, 165.0) | 160.0 (158.0, 164.0) | 0.142 |
| Weight, kg | 70.0 (64.5, 77.0) | 70.0 (62.7, 77.0) | 0.053 |
| Body mass index, kg/m² | 26.9 (24.9, 29.3) | 27.0 (24.4, 29.6) | 0.011 |
| Gravidity, n (%) |  |  | 0.138 |
| 1 | 5222 (44.0) | 370 (41.3) |  |
| 2 | 3341 (28.2) | 221 (24.7) |  |
| ≥3 | 3301 (27.8) | 305 (34.0) |  |
| Parity, n (%) |  |  | 0.170 |
| 0 | 7565 (63.8) | 548 (61.2) |  |
| 1 | 3834 (32.3) | 277 (30.9) |  |
| ≥2 | 465 (3.9) | 71 (7.9) |  |
| Multiple pregnancy, n (%) |  |  | 0.663 |
| No | 11731 (98.9) | 710 (79.2) |  |
| Yes | 133 (1.1) | 186 (20.8) |  |
| Gestational diabetes mellitus, n (%) |  |  | 0.064 |
| No | 9474 (79.9) | 692 (77.2) |  |
| Yes | 2390 (20.1) | 204 (22.8) |  |
| Intrahepatic cholestasis of pregnancy, n (%) |  |  | 0.319 |
| No | 11734 (98.9) | 829 (92.5) |  |
| Yes | 130 (1.1) | 67 (7.5) |  |
| Pregnancy-induced hypertension, n (%) |  |  | 0.286 |
| No | 11510 (97.0) | 807 (90.1) |  |
| Yes | 354 (3.0) | 89 (9.9) |  |
| Preeclampsia, n (%) |  |  | 0.537 |
| No | 11570 (97.5) | 732 (81.7) |  |
| Yes | 294 (2.5) | 164 (18.3) |  |
| Thyroid disease, n (%) |  |  | 0.048 |
| No | 10985 (92.6) | 818 (91.3) |  |
| Yes | 879 (7.4) | 78 (8.7) |  |
| Scarred uterus, n (%) |  |  | 0.050 |
| No | 9866 (83.2) | 728 (81.2) |  |
| Yes | 1998 (16.8) | 168 (18.8) |  |
| Maternal obesity, n (%) |  |  | 0.114 |
| No | 11332 (95.5) | 832 (92.9) |  |
| Yes | 532 (4.5) | 64 (7.1) |  |
| Group B Streptococcus infection, n (%) |  |  | 0.201 |
| No | 11079 (93.4) | 874 (97.5) |  |
| Yes | 785 (6.6) | 22 (2.5) |  |
| Vaginitis, n (%) |  |  | 0.038 |
| No | 11664 (98.3) | 885 (98.8) |  |
| Yes | 200 (1.7) | 11 (1.2) |  |
| Uterine fibroids, n (%) |  |  | 0.068 |
| No | 11271 (95.0) | 837 (93.4) |  |
| Yes | 593 (5.0) | 59 (6.6) |  |
| Cardiac arrhythmia, n (%) |  |  | 0.031 |
| No | 11722 (98.8) | 882 (98.4) |  |
| Yes | 142 (1.2) | 14 (1.6) |  |
| In vitro fertilization, n (%) |  |  | 0.387 |
| No | 11146 (93.9) | 730 (81.5) |  |
| Yes | 718 (6.1) | 166 (18.5) |  |
| Hepatitis B virus infection, n (%) |  |  | 0.032 |
| No | 11495 (96.9) | 863 (96.3) |  |
| Yes | 369 (3.1) | 33 (3.7) |  |
| Neutrophil count, ×10⁹/L | 6.5 (5.3, 8.1) | 7.3 (5.6, 9.7) | 0.309 |
| Lymphocyte count, ×10⁹/L | 1.5 (1.2, 1.8) | 1.5 (1.2, 1.9) | 0.032 |
| Hemoglobin, g/L | 120.0 (112.0, 128.0) | 117.0 (107.0, 126.0) | 0.237 |
| C-reactive protein, mg/L | 2.5 (2.5, 3.9) | 2.5 (2.5, 5.7) | 0.188 |
| Retinol-binding protein, mg/L | 34.6 (29.7, 39.8) | 32.2 (26.3, 38.4) | 0.244 |
| Prealbumin, mg/L | 202.7 (179.5, 227.1) | 191.2 (160.1, 220.6) | 0.307 |
| α-L-fucosidase, U/L | 51.2 (43.8, 59.3) | 49.3 (41.0, 59.2) | 0.072 |
| Leucine aminopeptidase, U/L | 240.1 (190.1, 300.5) | 222.3 (162.7, 301.5) | 0.116 |
| Cholinesterase, U/L | 5511.5 (4866.0, 6180.0) | 5206.0 (4512.3, 5972.3) | 0.243 |
| Total bilirubin, μmol/L | 8.0 (6.6, 9.9) | 7.5 (6.1, 9.6) | 0.038 |
| Direct bilirubin, μmol/L | 2.1 (1.7, 2.6) | 2.0 (1.6, 2.5) | 0.051 |
| Total protein, g/L | 63.3 (60.3, 66.3) | 61.2 (56.8, 65.0) | 0.467 |
| Albumin, g/L | 35.8 (33.9, 37.4) | 34.6 (32.0, 36.5) | 0.459 |
| Alanine aminotransferase, U/L | 9.8 (7.7, 13.0) | 11.70 (8.5, 18.1) | 0.240 |
| Aspartate aminotransferase, U/L | 17.0 (14.7, 20.0) | 18.1 (15.2, 23.3) | 0.156 |
| Total cholesterol, mmol/L | 6.4 (5.7, 7.3) | 6.3 (5.6, 7.3) | 0.009 |
| Triglycerides, mmol/L | 3.3 (2.6, 4.4) | 3.4 (2.6, 4.5) | 0.033 |
| High-density lipoprotein cholesterol, mmol/L | 2.2 (1.9, 2.5) | 2.15 (1.9, 2.4) | 0.144 |
| Low-density lipoprotein cholesterol, mmol/L | 4.1 (3.5, 4.8) | 4.0 (3.4, 4.9) | 0.054 |
| Apolipoprotein A1, g/L | 2.2 (2.0, 2.4) | 2.1 (1.9, 2.4) | 0.148 |
| Apolipoprotein B, g/L | 1.3 (1.1, 1.5) | 1.3 (1.1, 1.5) | 0.084 |
| Lipoprotein(a), mg/L | 77.9 (35.1, 171.4) | 103.8 (40.5, 210.1) | 0.148 |

**Table S7.** AUCs with 95% confidence intervals and pairwise DeLong comparisons for candidate models in the internal test set

| **Model** | **AUC** | **95% CI** | **DeLong *P* value vs LightGBM** |
| --- | --- | --- | --- |
| Logistic regression | 0.811 | 0.781–0.840 | 0.002 |
| LASSO logistic regression | 0.811 | 0.782–0.839 | 0.002 |
| Random forest | 0.831 | 0.804–0.858 | 0.140 |
| MLP | 0.806 | 0.777–0.835 | 0.001 |
| LightGBM | 0.842 | 0.817–0.867 | Reference |
| XGBoost | 0.837 | 0.811–0.863 | 0.273 |

**Table S8.** Sensitivity analysis of LightGBM model performance with and without LASSO-based feature selection

| **Model** | **Predictor set** | **Internal test AUC** | **95% CI** |
| --- | --- | --- | --- |
| Primary LightGBM | 37 LASSO-selected predictors | 0.842 | 0.817–0.867 |
| All-predictor LightGBM | All 42 candidate predictors | 0.847 | 0.823–0.871 |

**Table S9.** Net benefit and intervention trade-offs at representative threshold probabilities

| **Threshold probability** | **Intervention trade-off** | **Net benefit: model** | **Net benefit: classify all** | **Net benefit: classify none** | **Net reduction in unnecessary interventions per 100 women vs. classify all** |
| --- | --- | --- | --- | --- | --- |
| 5% | 19:1 | 0.044 | 0.041 | 0 | 4.4 |
| 10% | 9:1 | 0.007 | -0.012 | 0 | 16.9 |
| 15% | 5.7:1 | -0.021 | -0.071 | 0 | 28.6 |

**Table S10.** Sensitivity analyses of alternative risk stratification thresholds in the prospective validation cohort

| **Strategy** | **Low-risk cutoff** | **High-risk cutoff** | **Low risk, n/events (%)** | **Intermediate risk, n/events (%)** | **High risk, n/events (%)** | **RR: high vs. low (95% CI)** | ***P* for trend** |
| --- | --- | --- | --- | --- | --- | --- | --- |
| Top 10% / Bottom 50% | ≤50th percentile | ≥90th percentile | 4351/110 (2.5%) | 3417/220 (6.4%) | 1192/470 (39.4%) | 15.60 (12.80–19.00) | <0.001 |
| Top 15% / Bottom 50% (primary) | ≤50th percentile | ≥85th percentile | 4351/110 (2.5%) | 3003/179 (6.0%) | 1606/511 (31.8%) | 12.59 (10.33–15.34) | <0.001 |
| Top 20% / Bottom 50% | ≤50th percentile | ≥80th percentile | 4351/110 (2.5%) | 2499/138 (5.5%) | 2110/552 (26.2%) | 10.35 (8.49–12.61) | <0.001 |
| Top 15% / Bottom 40% | ≤40th percentile | ≥85th percentile | 3508/83 (2.4%) | 3846/206 (5.4%) | 1606/511 (31.8%) | 13.45 (10.75–16.83) | <0.001 |
| Top 15% / Bottom 60% | ≤60th percentile | ≥85th percentile | 5204/150 (2.9%) | 2150/139 (6.5%) | 1606/511 (31.8%) | 11.04 (9.28–13.13) | <0.001 |

**Table S11.** Global feature importance of the LightGBM model based on SHAP analysis

| **Rank** | **Feature** | **Mean absolute SHAP value** |
| --- | --- | --- |
| 1 | Neutrophil count (NEU#) | 0.393 |
| 2 | Retinol-binding protein (RBP) | 0.319 |
| 3 | Leucine aminopeptidase (LAP) | 0.316 |
| 4 | Alanine aminotransferase (ALT) | 0.309 |
| 5 | Weight | 0.295 |
| 6 | Maternal age (Age) | 0.217 |
| 7 | Multiple pregnancy | 0.158 |
| 8 | Prealbumin (PA) | 0.148 |
| 9 | Direct bilirubin (DBIL) | 0.134 |
| 10 | Albumin (ALB) | 0.133 |
| 11 | Group B Streptococcus (GBS) infection | 0.129 |
| 12 | Preeclampsia | 0.082 |
| 13 | Lipoprotein(a) (Lp(a)) | 0.080 |
| 14 | Alpha-L-fucosidase (AFU) | 0.079 |
| 15 | Total bilirubin (TBIL) | 0.076 |
| 16 | Total protein (TP) | 0.068 |
| 17 | Cholinesterase (CHE) | 0.057 |
| 18 | Hemoglobin (HGB) | 0.046 |
| 19 | Triglycerides (TG) | 0.047 |
| 20 | Apolipoprotein A1 (ApoA1) | 0.040 |
| 21 | In vitro fertilization (IVF) | 0.035 |
| 22 | Lymphocyte count (LYM#) | 0.032 |
| 23 | Low-density lipoprotein cholesterol (LDL) | 0.025 |
| 24 | Intrahepatic cholestasis of pregnancy (ICP) | 0.024 |
| 25 | Gravidity = 2 | 0.027 |
| 26 | Total cholesterol (TC) | 0.018 |
| 27 | Parity ≥ 2 | 0.016 |
| 28 | Obesity | 0.013 |
| 29 | Apolipoprotein B (ApoB) | 0.012 |
| 30 | Hepatitis B virus (HBV) infection | 0.010 |
| 31 | Parity = 1 | 0.005 |
| 32 | Gestational diabetes mellitus (GDM) | 0.004 |
| 33 | Pregnancy-induced hypertension (PIH) | 0.001 |
| 34 | Thyroid disease | 0 |
| 35 | Vaginitis | 0 |
| 36 | Uterine fibroid | 0 |
| 37 | Cardiac arrhythmia | 0 |
